# Wild capuchin monkeys use stones and sticks to access underground food

**DOI:** 10.1038/s41598-024-61243-8

**Published:** 2024-05-06

**Authors:** Tatiane Valença, Gabriela Oliveira Affonço, Tiago Falótico

**Affiliations:** 1 University of São Paulo, São Paulo, Brazil; 2 Capuchin Culture Project, Neotropical Primates Research Group, São Paulo, Brazil; 3 Department for the Ecology of Animal Societies, Max Planck Institute of Animal Behavior, Konstanz, Germany; 4 Technological Primates Research Group, Max Planck Institute for Evolutionary Anthropology, Leipzig, Germany

**Keywords:** Animal behaviour, Anthropology

## Abstract

Primates employ different tools and techniques to overcome the challenges of obtaining underground food resources. Humans and chimpanzees are known to tackle this problem with stick tools and one population of capuchin monkeys habitually uses stone tools. Although early hominids could have used stones as digging tools, we know little about when and how these could be useful. Here, we report a second primate population observed using stone tools and the first capuchin monkey population to habitually use the ‘stick-probing’ technique for obtaining underground resources. The bearded capuchin monkeys (*Sapajus libidinosus*) from Ubajara National Park, Brazil, use ‘hands-only’ and ‘stone-digging’ techniques for extracting underground storage organs and trapdoor spiders. Males also use ‘stick-probing’ and ‘stone-stick’ techniques for capturing trapdoor spiders. Tool use does not increase success in obtaining these resources. Stone-digging is less frequent in this population than in the only other known population that uses this technique. Females use stones in a lower proportion of their digging episodes than males in both populations. Ecological and cultural factors potentially influence technique choice and sex differences within and between populations. This population has a different pattern of underground food exploration using tools. Comparing this population with others and exploring the ecological and cultural factors under which capuchin monkeys employ different tools and techniques will allow us to better understand the pressures that may have shaped the evolution of those behaviors in primates.

## Introduction

Our ancestors might have used different tools and techniques to overcome the challenges of obtaining underground food. Ethnographic studies show that contemporary hunter-gatherers use digging stick tools to obtain tubers and roots, and also to dig out burrowing animals^1,2^. Hidden underground food items such as underground storage organs, harvested during periods of scarcity in the dry savannah, are thought to have been an important fallback food in human evolution^3,4^. Archaeological evidence shows that the earliest human digging stick tools found are as recent as 3500 years BP^5^, but they might have used these tools before since organic materials are difficult to preserve in the archaeological record. Bones had also been used by early hominids, supposedly to excavate termites^6,7^.

Stones are suggested as a potential material for digging tools that could have been used by early hominins^8,9^, especially pointed natural rocks^5^. These objects are thought to have been useful during the dry season when the soil in the savannah toughens, making digging likely inefficient with sticks that were not fire-hardened^10^. Although suggested as potential good materials, stones as excavation tools were never studied in hominids. We need to learn more about when and how they might have used them, or how to recognize them in the archeological record, in order to understand the origins of digging tools in human lineage.

Nonhuman primates also use different tools to access underground resources and studying them can help to fill this gap in human evolution. Chimpanzees (*Pan troglodytes*) and robust capuchins (*Sapajus* spp.) can use sticks and bones to excavate food in experimental settings^10–12^. In the wild, chimpanzees use sticks to dig insect nests^13^ and to dig underground storage organs^14^. They also use sequential sticks to perforate ground termite chambers and fish for termites^15^.

The use of digging stone tools to obtain underground food was reported in only one population of primates, the bearded capuchins (*Sapajus libidinosus*) from Serra da Capivara National Park (SCaNP). This population lives in a Brazilian dry savannah area, and the capuchins use stone tools to access underground storage organs, roots, trapdoor spiders, and other arthropod nests^16–18^. This was also the only capuchin population observed to habitually modify and use sticks as probes to dip for honey and expel prey (such as lizards, bees, and scorpions) from rock crevices and trunks^17,19^, and occasionally for other resources.

Here, we report another population of bearded capuchin monkeys (*S. libidinosus*) that uses stones for digging, and this is the first population observed to use the ‘stick-probing’ technique to obtain underground food. These monkeys live in Ubajara National Park (Brazil), a much wetter savannah area, and are already known for using hammerstones for palm nut-cracking^20,21^. We describe two techniques used for extracting underground storage organs and four techniques used for the capture of trapdoor spiders. We compare the techniques across capuchin populations and with those of chimpanzees, discussing the implications for understanding the evolution of tools to obtain underground food in the primate lineage.

## Results

We followed a group of 31 monkeys for 21 months, recording episodes of digging and probing to obtain underground food resources. We observed 214 episodes of digging for underground food, with or without tools. The total digging rate was 0.39 episodes/100 h/individual. The capuchins excavated two resources: underground storage organs (USOs) and trapdoor spiders. The USOs are tuberous roots with reddish-brown skin that the monkeys peel with their hands and teeth before consuming the flesh (see a picture of these USOs in Fig. S1). The trapdoor spiders excavated were *Idiops sertania* and *Neodiplothele* sp. (see pictures of these spiders and their burrows in Fig. S2). The capuchins usually remove the operculum covering the spider burrow and excavate it, accessing and consuming the spider and the ootheca. They excavated more USOs (49%) than spider burrows (19%). Most USO (96%) and spider burrow (55%) digging episodes occurred in hills, and 45% of spider burrow digging episodes took place on riverbanks. See pictures of the hills and the riverbanks in Fig. S3. We were unable to determine the food target in 32% of the digging episodes, either because we could not reach certain steep areas or because we were unable to identify the leftovers.

### Stone tools

**Figure 1:**
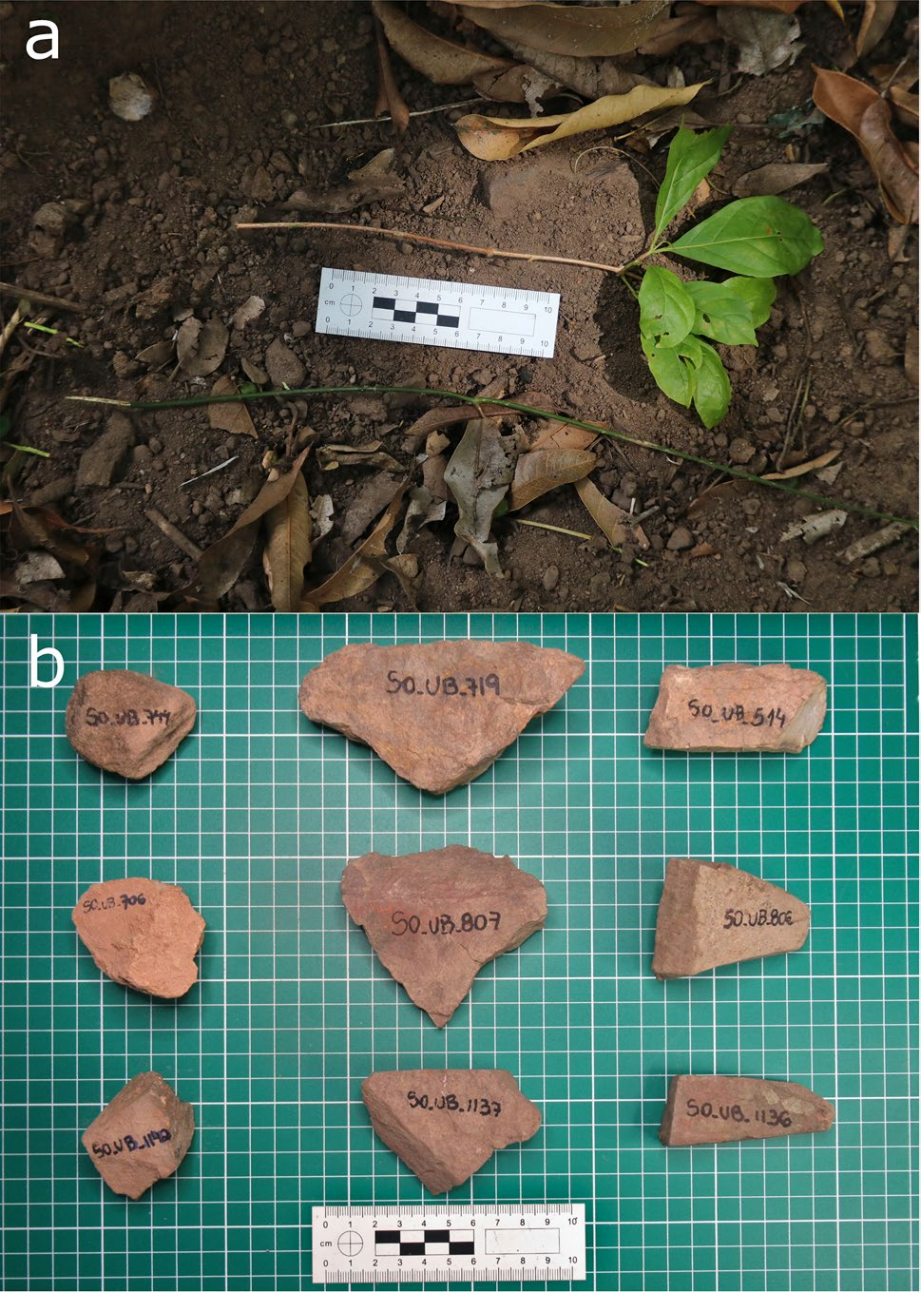
Tools used by the capuchin monkeys at Ubajara National Park. (**a**) Stick tools used to probe trapdoor spider; (**b**) digging stones used to excavate underground food. Scale: 10 cm.

The monkeys used digging stone tools (Fig. 1) in 51.4% of digging episodes. See examples of monkeys excavating with stone tools in Video S1. The digging with stone tools rate was 0.20 episodes/100 h/individual. The overall proportion of success was 31.8%. The digging with hands-only rate was 0.19 episodes/100 h/individual, with a proportion of success of 40.4%. Stone tools were used to aid in digging in 59% of USOs and in 48% of spider burrow digging episodes (Table 1). When not using tools—the ‘*hands-only*’ technique—, the monkeys used just their hands to remove soil from the substrate and stones embedded in the soil and pull the exposed root of the USOs with their teeth. For trapdoor spiders, they broke the soil around the burrow with their hands, reducing the burrow length and inserting their fingers to access and consume the spider and/or its ootheca. When using stone tools—the ‘*stone-digging’* technique—, the monkeys pounded the tool against the soil to break it loose, facilitating the extraction of USOs or reducing the length of the spider burrows. The monkeys used the digging stone tools in a higher proportion of the digging episodes in hills (59%) than in those in riverbanks (24%). Females used digging stone tools in a lower proportion of their digging episodes than males (Table 2). This difference between sexes was significant (Fisher’s exact test, p < 0.001; juveniles were not included in this analysis). This finding is also supported by a supplementary analysis (see Tables S5–S8 and a discussion of the advantages and limitations of different methods in the Supplementary Material). The sex did not influence excavation success with or without tools (see Table S1 for statistical results). We collected and measured 50 digging stone tools (see details on dimensions for each target resource in Table S2). Most of them are made of sandstone materials and are smaller and lighter (average weight 128 g) than the pounding tools used for palm nut cracking (average weight 1142 g) by the same population^20^.

**Table 1:** Digging events targeting USOs and spider burrows, N = 145.

	Target	
	USO	Spider burrow
Total episodes	105	40
Digging with tools	59% (62/105)	48% (19/40)
Digging without tools	41% (43/105)	53% (21/40)
Success rate with tools	34% (21/62)	58% (11/19)
Success rate without tools	47% (20/43)	67% (14/21)
Hole depth (cm)^a^	4.8 ± SD 2.7 (0.5–15)	6.1 ± SD 2.5 (2–12.5)

Only episodes with resource identification are analyzed. All sexes and ages are included.

We could not determine the food target in 32% of the digging episodes.

^a^In this case, N = 71 for USO and N = 29 for spider burrow, since steepness prevented us from reaching some of the excavated areas to measure the hole depth or we were unsure about which hole was excavated.

**Table 2:** Digging events by sex, N = 175.

	Males	Females
Total episodes	133	42
Digging with tools	65% (86/133)	10% (4/42)
Digging without tools	35% (47/133)	90% (38/42)
Success with tools	34% (29/86)	50% (2/4)
Success without tools	40% (19/47)	50% (19/38)

All food resources are included in this analysis. Juveniles were not included in this analysis.

### Stick tools

We recorded 40 episodes of capuchin monkeys using sticks on the ground. The probing tool use rate on the ground was 0.07 episodes/100 h/individual, with an overall success rate of 42.5%. Dry twigs were obtained from the ground, and fresh twigs from surrounding bushes, without removing leaves. The monkeys used more than one stick tool in 47.5% of the episodes, totaling 74 sticks used on the ground. In 32 episodes they used those sticks for foraging spider burrows. This represents 37.2% of all 86 episodes in which they foraged in spider burrows. We were unable to determine the food target in 8 episodes they used sticks on the ground, either because we could not reach certain steep areas or because we were unable to identify the leftovers.

The use of sticks on the ground was observed only in adult and subadult males in the Sertão Group and in the other groups living in the area, from which we opportunistically observed three episodes performed by two different individuals. In the sampled capuchin group (Sertão), probing on the ground was observed in 2 adults and 4 subadult males. One of these subadults observed using a stick on the ground left the group before 2022. One young adult male was not observed using stick tools. Another adult male left the group before the second dry period, and he was not seen probing on the ground, although he was seen using a stick tool in a rock crevice in the new group. In sum, 7 out of 8 males that integrated the Sertão Group at some point in the sampled period were seen using sticks for some purpose. Juveniles were not seen using sticks, although they observed stick tool use, manipulated sticks, and inspected the holes and the spider burrow’s *opercula*.

Stick tools were used more in riverbanks (63%) than in hills. We collected and measured 30 stick tools used on the ground (Fig. 1). The tools had an average total length of 29.4 cm ± SD 12.6 (8.5–61.3), and average thickness of 2.9 mm ± SD 0.12 (1.5–7). See Table S3 for a comparison between stick tool dimensions in UNP and SCaNP.

### Techniques used for spider capture

We observed four techniques capuchins used to access and capture the trapdoor spiders. The first two, *hands-only* and *stone-digging* techniques were described above for extraction of USOs. For the spider, the monkeys also used the ‘*stick-probing*’, when they remove the *operculum* using their hands, insert the twigs into the burrow, and energetically shake the tool in a side-to-side motion, forcing the spider to get out and allowing the capture, or extracting the ootheca from the spider burrow. See examples of this technique in Video S1. This technique was observed only in males. Adult males sometimes hold the probe in one hand and place the other hand on the side of the burrow, apparently to prevent the spider from falling and running away. After the extraction of a spider or an egg sac, they usually reinsert the sticks, sometimes pulling out the remaining food resource.

**Figure 2:**
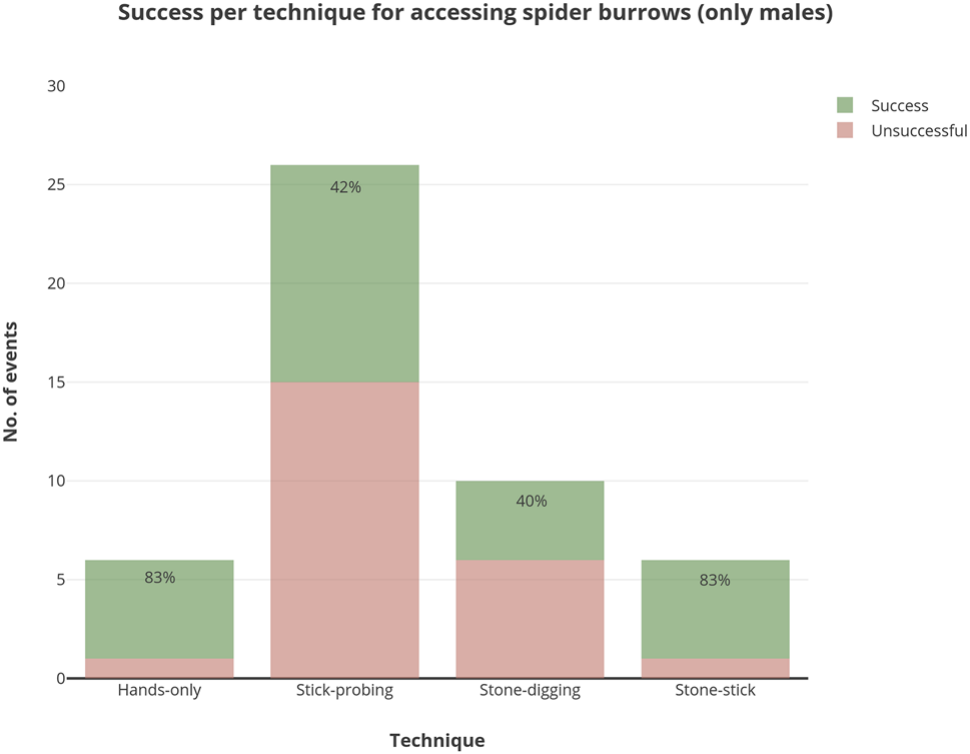
Number of episodes and success per technique used by the capuchin monkeys for accessing spider burrows (N = 48). Stone-stick refers to episodes when both stones and sticks were used. Females and juveniles were not included.

The last technique observed and used only in spider burrows was the ‘*stone-stick*’, when stone tools were apparently used to reduce burrow depth, possibly facilitating the use of sticks to probe or extract spiders and/or ootheca. We observed the sequential use of stones and sticks in six episodes, performed exclusively by males. See an example of this technique in Video S1. There was no clear pattern in the choice of the first tool. The tools were exchanged during the episode, but the last tool used was always a stick. The success of the different techniques used by males for accessing spider burrows are compared in Fig. 2. Tool use did not increase success in any category (see Table S4 for details on statistical results).

### Comparing dry and wet seasons

The total digging rate was higher during the dry season (0.48 episodes/100 h/individual) in comparison to the wet season (0.30 episodes/100 h/individual). However, during the wet season we observed them successfully exhibiting a fifth technique to obtain USOs, the ‘teeth-only’, pulling the exposed root of the USOs with their teeth without using hands or stones. As we were focused on comparing digging behavior (i.e. removing the soil using their hands, with or without the aid of stones), we did not record this technique. Thus, although they excavated more during the dry season, we cannot affirm that they forage more underground resources during this period.

In the wet season, the general rates of digging using hands-only (0.16 episodes/100 h/individual) were similar to digging with stones (0.14 episodes/100 h/individual). However, the rates of extracting these resources without stones in this period may be higher, as we did not consider the ‘teeth-only’ technique. On the other hand, during the dry season, the rates of digging using stones were slightly higher (0.26 episodes/100 h/individual) than without stones (0.22 episodes/100 h/individual). The use of sticks (together or not with stones) for acquiring underground resources was observed exclusively during the dry season, except for one case during the wet season in which we could not identify the target resource. The probing rate was higher in the dry season (0.15 episodes/100 h/individual) than in the wet season (< 0.01 episodes/100 h/individual).

Regarding the spider burrows, males and females foraged more on this resource during the dry season and used more techniques (see a comparison of techniques used in each season in Fig. S4). Males also foraged more in spider burrows than females. The real size of this difference is probably higher because we did not consider the contact time with each sex for the calculation and there are more females in the group. For USOs, males and females used stone-digging and hands-only techniques in similar proportions during dry and wet seasons (see a comparison of techniques used in each season in Fig. S5). Since we did not record the ‘teeth-only’ technique, those proportions might change when this behavior is included in future calculations.

### Ecological sampling

The measurement of soil cohesion values with the pocket penetrometer was not feasible (see “Methods”). However, it comparatively indicated a high soil cohesion in UNP in the areas Sertão Group excavated.

The measurement with the drop penetrometer (see “Methods”) in SCaNP resulted in an average soil penetration of 6.24 cm ± SD 1.13 (3.5–9.5); and in UNP it was 4.68 cm ± SD 1.03 (2.0–8.5), indicating a higher soil cohesion in UNP (t-test: t = 8.049, df = 83, p < 0.001) in the home range area of Sertão Group.

For superficial stones, our sampling indicated an average of 36.9 stones/m^2^ in the home range area of the Sertão Group (UNP). This is more than two times higher the average found in Pedra Furada Group living area^22^ (SCaNP, 16.8 stones/m^2^).

## Discussion

In this study, we reported the use of stones and sticks to access underground food in a population of bearded capuchin monkeys living in the Ubajara National Park (UNP), a wetter Brazilian savannah. We described the use of hands-only and stone-digging techniques to access underground storage organs. In addition to these two techniques, this population also uses stick-probing and stone-stick techniques to extract trapdoor spiders from the ground.

The use of stones and sticks as tools to obtain food is within the behavioral repertoire of both UNP and Serra da Capivara National Park (SCaNP) populations. However, these populations present differences in stone-digging rates. We found a digging stone tool use rate in UNP approximately six times lower than the rate found in SCaNP (1.25 episodes/100 h/indiv.^18^). One possible explanation for this difference is a higher cohesion of the soil in UNP that could make soil exploration more difficult and reduce the rates of digging in general, with or without tools. Our ecological sample showed higher relative cohesion of the soil and a higher number of superficial stones in Sertão Group (UNP) than in Pedra Furada (SCaNP) living area. Unfortunately, hands-only digging rates in SCaNP have not yet been assessed. Differences in food preferences and availability of resources between sites are alternative explanations that could reduce general rates. The areas the monkeys excavated in UNP also posed limitations in our study, since sometimes we lost the group while following them in hills. Using remote sensing technology^23^ to track the monkeys and calculate more precisely the time they spend in hills and sampling the availability of the USO’s trees can provide a better estimate of the size of these differences in the rates between the sites.

These populations also present differences in techniques used to access underground food. Tool use in capuchin monkeys from SCaNP has been studied for over two decades^16,17,19,24^ and trapdoor spiders are the most excavated resource besides USOs in SCaNP^18^; however, the use of sticks for spiders was recorded in just five events (i.e., five sticks used) during this time period^25^. More precisely, these five sticks were used in only three episodes specifically for underground trapdoor spiders (T. Falótico, unpublished data). In these three episodes, they employed the ‘stone-stick’ technique and were successful in just one. Stick-probing technique alone was never observed there. Moreover, in SCaNP the monkeys always remove branches and lateral leaves when using sticks^19^. Based on this, we can conclude that the use of sticks for trapdoor spiders and sticks with leaves constitutes a behavioral variation between these populations.

Differences in techniques for extracting trapdoor spiders may be caused by two non-exclusive ecological factors. Firstly, differences in soil properties may cause the stone-digging technique to be less successful in UNP, requiring the use of sticks for trapdoor spiders in certain contexts. Although we do not have proper measurements, the riverbanks and the hills in UNP seem to have stonier and tougher soil compared to SCaNP terrain. In relation to the living area of the sampled groups, the soil in UNP has a higher relative cohesion and a higher number of superficial stones than in SCaNP, evidence that favors this hypothesis. This is also supported by the pattern observed in the stone-stick technique: even after digging spider burrows with stones, the last tool used was always a stick—and this stone-stick technique is more successful than the stone-digging alone. Secondly, the capuchin monkeys forage on a different trapdoor spider genus^18^. Possible differences in the spider’s behavior (e.g., spider grasping itself in the tunnel or running away when disturbed) may lead to stick tools being less effective in SCaNP.

However, even if ecological factors are causing the behavioral variation between UNP and SCaNP, we cannot rule out the possibility that this technique is a cultural trait in UNP. Cultural traits are acquired by socially biased learning mechanisms, and environmental factors can affect the emergence or maintenance of these cultural traits^26–28^. At UNP, the immatures closely observed proficient individuals, manipulated stones, sticks, and the *operculum*, and explored spider burrows. Moreover, the stone-stick technique may be considered a toolset, which is defined by the sequential use of tools with different functions to achieve a goal^29^. Toolsets or multi-step extractive processes are not commonly observed in wild animals^30^, are less likely to be independently reinvented, and are possible precursors of a cumulative culture^31^. Although used at a lower rate and in a not well-defined sequence, it is noteworthy that this technique presents higher success rates than stone-digging and stick-probing separately, elements that constitute this more complex stone-stick technique. These suggest that socially biased learning mechanisms may be involved in the maintenance of different tool use techniques in UNP, as already suggested for the development of probe tool use in the monkeys of SCaNP^24^ and as demonstrated for the use of stone tools for nut-cracking in capuchins^32,33^.

Females used digging tools in a lower proportion of their digging episodes than males and did not use sticks. Sex differences have been also observed in SCaNP population, such as adult females in SCaNP using fewer stone tools in their digging episodes than males and not using sticks for foraging^18,19^. Those sex differences can have several explanations, such as lack of interest or lower ability. An absence of interest in underground food resources cannot explain sex differences because females obtain the same resources using the hands-only technique. A lower ability of females to use these tools cannot explain sex differences either. The males were not more successful in digging with stones in comparison to females both in UNP and in SCaNP^18^, and females are able to use sticks in captivity^34^.

Another factor that apparently explain those sex difference is the success of the techniques depending on the ecological context. In Fazenda Boa Vista (FBV), another site where bearded capuchin monkeys excavate USOs, both sexes use exclusively the hands-only technique in a similar proportion (males: 43%; females: 57%; proportions calculated with raw data available in^35^), there is no significative difference in excavation between the sexes, and the success is high (82.4%)^35^. In contrast, in UNP the hands-only technique for USOs has a success of 47% and for spider burrows 67%. The overall success of stone-digging and stick-probing in obtaining underground resources in UNP and SCaNP are also lower (stone-digging, UNP: 31.8%, SCaNP: 38%^18^; stick-probing, UNP: 42.5%). Females are suggested to be more sensitive to food rewards and less motivated to use tools when there is no considerable increase in food gain^19^. The low success of tool techniques used for underground resources in UNP when compared to techniques without tools support this idea, as females would be more conservative in their foraging strategies if the alternative does not increase food gain.

It is intriguing that the use of tools did not increase overall success in obtaining underground food resources. However, we could not evaluate other efficiency parameters, such as whether they use tools to obtain larger resources (e.g., bigger USOs) or to reduce the duration of excavation. Differences in ecological conditions within and between sites may also be a possible explanation for both the use and choice of different tools and techniques. We hypothesize that a more resistant and stonier soil can pose more challenges in extracting underground resources, leading the monkeys to choose a different technique (hands-only, stone-digging, stick-probing, or stone-stick). We predict that capuchin monkeys use hands-only in looser soil and stone-digging in compacted and tougher soil. In UNP, we observed the individuals struggling when attempting to access spider burrows in extremely stony soil, between embedded stones in hills and riverbanks that were difficult to remove. A thin stick tool could be efficient in reaching spiders in stony soil when stone-digging is useless. Moreover, we also hypothesize that capuchin monkeys actively choose stone tool positions and that this increases efficiency when digging in tough soil. In UNP, we observed the monkeys apparently adjusting the position of digging stone tools, using the distal and angular areas of the stones (see an example of this behavior in Video S1). Indirect evidence of digging tools' wear marks in SCaNP support this hypothesis^8^.

Considering the tough soil’s hypothesis, it would be expected that the monkeys use stone and thin stick tools relatively more in the dry season—when the soil tends to harden—than during the wet season. We do not have evidence that supports this use for the stones, since we found just a slight difference in the use of stones between seasons. We also could not rule out that they use other techniques without tools in the wet season, such as the extraction of USOs with teeth (‘teeth-only’, without using hands), that seem to occur only during the wet season and was not recorded in the present study. They used, however, three techniques involving tools to forage trapdoor spiders during the dry season, particularly techniques involving sticks (stick-probing and stone-stick), which a priori favor this hypothesis. They also foraged more spiders during the dry season, which could be explained by a myriad of possibilities: this may be a fallback food, provide certain necessary nutrient, be more accessible due to the lower water level of the river, or be more available during this season. Another possibility is that the reproductive period of these spiders occurs in the dry season, then the females could be larger or have egg sacs within their burrows, increasing the food return. Although we observed them successfully extracting egg-sacs using hands-only and stone-digging, we could not evaluate whether sticks were more efficient for this specific task.

Chimpanzees seem to adjust their strategies for obtaining underground food depending on ecological conditions. In Loango, the soil is tougher during the dry season^36^ and they use a larger behavioral repertoire to excavate bee nests in tough soil^13^. In Ugalla, all excavated sites of USOs were recorded during the rainy season, and the authors suggest that the soil is overly tough during the dry season, preventing the chimpanzees from excavating^14^. However, chimpanzees did not use tools more frequently in compacted than in loose soil in experimental settings^10^. The authors themselves draw attention to a potential insufficient difference in compactness between the two soil conditions that may not have been adequate to test the chimpanzees' choice. Thus, the degree of compactness may matter when choosing stone-digging techniques. Future empirical and observational studies with nonhuman primates should include the measurement of soil compactness in their protocols to evaluate this possibility. Further exploration on more subtle differences in soil compactness between areas of the same site within the same season are also necessary.

The use of digging tools in hominids has been suggested to be influenced by ecological conditions. Modern humans use digging sticks to excavate tubers that are 25–50 cm deep in compacted and stony soil^2^. Motes-Rodrigo et al.^10^ suggested that using sticks not hardened by fire should have been inefficient in the extremely hard soil of savannah during the dry season and that early hominins should have used stones instead of sticks to excavate USOs for this reason. Pointed natural rocks are hypothesized as materials that humans could have used for digging that could aid in piercing and breaking up the tough soil^5^. Nonhuman primates may shed light on these hypotheses. Since Platyrrhini diverged from Catarrhini around 40 million years ago, digging tool use in capuchins, chimpanzees, and humans cannot be homologous. Investigating in-depth the ecological hypothesis we raised in this study in both capuchins and chimpanzees is fundamental to evaluate possible evolutionary convergences, as well as differences in how humans and other primates respond to environmental challenges when accessing underground food. These can also potentially help us in predicting where and when we could expect to find different digging tools in the archeological record.

In conclusion, our study showed that capuchin monkeys can employ a multitude of behaviors in the wild to solve the issue of underground food acquisition and that these techniques vary between sexes and sites. We discussed several ecological factors that might be involved in these variations and in the choice of different techniques, possibly interacting with local groups' cultural repertoires. By exploring the ecological factors under which capuchin monkeys employ hands, stones, or sticks to obtain underground food and understanding how they choose and use these tools in each situation, we will better comprehend the ecological pressures that may have shaped the emergence of different digging tools and techniques in the primate lineage.

## Methods

### Study site and subjects

This study was conducted in Ubajara National Park (UNP, State of Ceará, Brazil). The park is within Caatinga Biome (dry savannah), although is a much more humid area compared to Serra da Capivara National Park. The altitude varies between 300 and 900 m. This difference in altitude causes marked differences in temperature, precipitation, and vegetation. The highland is colder (average temperature: 26.1 °C), has a higher annual rainfall (average rainfall: 1459 mm) and features tropical wet forest vegetation. In contrast, the lowland is hotter (28.2 °C), drier (average rainfall: 939 mm), and presents vegetation composed of dry forest and stepic savannah^37,38^. The wet season occurs from January to June, and the dry season occurs from July to December^20^. Plants species reported to be dug with tools by SCaNP population (farinha-seca, *Combretum glaucocarpum*, and louro, *Ocotea* sp.) are present in UNP^18^.

The bearded capuchin monkeys (*Sapajus libidinosus*) living in this location are known to use stone tools as hammers and anvils to crack open encased resources, mostly palm nuts^20,21^. The Sertão Group was composed of 31 individuals for most of the period sampled, 3 adult males (more than 7 years), 3 subadult males (5–7 years), 10 adult females (more than 5 years), 9 juveniles (2–5 years) and 6 infants (0–2 years). Female capuchin monkeys first conceive at 4.9 to 7 years old^39^ and are accounted as adults since then, even though they did not reach full adult body mass at this time^40^, not presenting the subadult phase as males. We combined adult and subadult males for the analyses, as done in other studies of digging behavior in capuchins^35,41^. In most analyses, we excluded infants and juveniles because individuals below five years are still learning to use tools and increasing proficiency in these tasks^18,24,42^. The ages were estimated based on morphological characteristics since the group was not studied before. We inferred age categories based on the conjunction of several characteristics: body size, tufts size, teeth size, number of teeth, integrity of teeth, scars, body integrity, fur color, strength in movements, pregnancy, and breastfeeding behavior. The group lives in the lowland (− 3.82656, − 40.89487), a drier and hotter area of the park. The Sertão Group’s range area comprises a valley with hills and both perennial and intermittent small streams. In the hills, the soil is dry and covered with slippery fallen leaves during the dry season, whereas it is moist and covered by green bushes during the wet season (thorny deciduous forest).

### Data collection

We followed the group from October 2021 to July 2023, from initial visual contact in the early morning until dusk or the loss of contact, totaling 1778.6 h of contact time (913.5 h during the wet season and 865.1 during the dry season).

We used “All Occurrences” sampling to record digging episodes, with or without tools, and stick tool use episodes on the ground. This was the same sampling method used in SCaNP for data collection^17,18^. All occurrences and ad libitum present limitations in calculating rates of behaviors since sampling may not be evenly distributed across individuals. However, they are commonly used to study tool use in capuchin monkeys because it tends to be an infrequent behavior^18,19,41,43^. We recorded most episodes filming them with a camcorder *Canon Vixia HF*, but we also used audio and written notes as records. In several cases it was not possible to video-record the episodes from the very beginning, because the terrain was steep and not easy to position the camera quickly. While filming, we also voice recorded all relevant information related to the episode in case it was not possible to see clearly or the event was not recorded from the beginning. When possible, we also video-recorded the area excavated after the episodes. We did not register episodes of digging for dry macaúba nuts (*Acrocomia aculeata*). Those are nuts they crack with stones^20^ and that accumulate on the ground around palm trees and nut-cracking sites. The monkeys often use their hands to remove the surface layer of the soil and access the buried nuts. We never observed the monkeys using digging stone tools in these macaúba digging episodes.

The data collection occurred in two phases. In the first phase (October/2021 to July/2022) we recorded digging behavior and the use of sticks while following the group, using audio or written notes to record additional data related to the episode. In the second phase (August/2022 to July 2023) we systematically followed one adult a day by month (focal individual) for collecting behavioral data for ongoing studies (see Fig. S6). During this phase, we also recorded All-Occurrences of digging and stick tool use performed by any individual of the group (focal or not). When we observed an episode, we also recorded date, time, individual identity, GPS location, target dug or probed, tools dimensions, tool weight, excavation depth, percent of canopy cover, distance from the nearest tree, and additional information about the area excavated in a digital form of *KoboCollect* App in a cellphone *Doogee Rugged S97 Pro*. Figure S6 shows a comparison between the two phases, detailing the behavioral and time sampling used, as well methodological advantages and limitations.

We collected digging stone tools and stick tools we could access and identify, measuring their weight and dimensions, using the same measurement methods used in previous work at SCaNP^41^. To calculate digging and tool use rates (episodes/100 h/individual), we summed up all the episodes and divided them by the number of contact hours and by the number of individuals in the group. We considered a successful episode when the food resource was extracted from the soil, even if the monkey did not consume it. To calculate digging and tool use rates per season and sex, we divided the number of episodes of each technique by the contact hours with the group during the corresponding season. We did not consider the contact hours with each sex. We used the months to define wet (January to June) and dry (July to December) seasons based on historical weather data^20^.

To try to assess absolute soil cohesion, we used a pocket penetrometer during the dry season of 2022 (Dial Geopocket Penetrometer, Fig. S6). According to the equipment protocol, one has to take the equipment between the thumb and the index, and lean the prod to the soil, and then push it gradually until the prod penetrates up to the notch on it. The protocol says to take 10 measures in the area around the monkeys extracted underground food and calculate the average measure. Even using the smaller prod (Ø6.4 mm), in several cases we exceeded the upper limit of the equipment (6 kg/cm^2^). The penetrometer was not indicating accurate measures and using it could damage the equipment. Moreover, the protocol requires a plain terrain, what was not the case for most areas the monkeys excavated and used sticks in UNP.

To compare capuchin monkey sites, we conducted plot samples. We recorded tracks using Gaia GPS App while following the Sertão Group from October 2021 to May 2022. In QGIS, we used these tracks to create a Minimum Convex Polygon and estimate Sertão Group home range area. We adjusted 40 plots of 110 × 110 m in this estimated home range area and randomly drew 3 points in each plot, totaling 120 points.

To evaluate relative soil cohesion, we sampled 1 point per plot (N = 40). We used a homemade “drop penetrometer”, using a conical metal weight with markings (1969.6 kg, 20 cm length, 4.96 cm diameter, Fig. S7) and a plexiglass tube (75 cm length, 5 cm internal diameter). In each point, we positioned the tube with one end on the ground and we inserted the metal weight in the tube, aligned with the tube upper end border. Then, we dropped the weight to the ground and checked how many centimeters it penetrated the soil. We removed the surface litter and avoided visible stones during the positioning. We took 10 measures and averaged them for each point. The weight penetrates more on less cohesive soil. The same procedure was performed in SCaNP in the same plots used in other study^22^, within the living area of Pedra Furada Group.

We used the same plots to estimate the number of superficial stones in UNP, sampling all 120 points. For each point, we used a 1 × 1 m square and counted all movable superficial stones with more than 3 cm, similar to the method used in another study in SCaNP^22^, which contains the data we used to compare with UNP.

### Data analysis

We used Generalized Linear Models (GLM) to examine the effect of the independent variables sex and target on the dependent variable success in digging (with or without stones; Table S1). In this case, we excluded episodes with unknown food targets and from juveniles due to the low number of juvenile females that excavated food. To evaluate the different male techniques in obtaining spiders, we used a GLM model with success as the dependent variable and the four different techniques (hands-only, stone-digging, stick-probing, stone-stick) as independent variables (Table S4). Females and juveniles were not included as they were not seen exhibiting all techniques. In all models where the dependent variable was “success”, we used a binomial distribution.

We used a Fisher’s exact test to compare sex differences between digging techniques. Juveniles were not included in the analysis because of the low number of female juveniles observed digging. To analyze relative differences in soil between sites, we used a Two-Sample t test. We performed a Shapiro–Wilk normality test before it to check whether data had a normal distribution. The analyses were conducted in R 4.3.2^44^ and we applied a significance level of 0.05 as a threshold for all tests.

### Ethical statement

The research was observational and complied with protocols approved by the Animal Research Ethical Committee of the School of Arts, Sciences and Humanities, University of São Paulo (CEUA/EACH 006/2021); and to Brazilian law under authorization from ICMBio (SISBIO #78349-1). This research adhered to the American Society of Primatologists (ASP) Principles for the Ethical Treatment of Non-Human Primates.

### Supplementary Information

Supplementary Information 1.

Supplementary Information 2.

Supplementary Video 1.

## Data Availability

The data used for the analysis of this paper is available as supplementary material.
