# Supplementary material for: Wild capuchin monkeys use stones and sticks to access underground food
Source: Sci Rep. 2024 May 6;14:10415. doi: 10.1038/s41598-024-61243-8 (PMC11074112; doi:10.1038/s41598-024-61243-8)
Supplement: Supplementary file 1 — Supplementary Information 1. [file 41598_2024_61243_MOESM1_ESM.pdf]

Digging data

Tool use	Stick	Date	Time	Lat	Long	Tool1	Tool2	Individual	Sex	Age	Behavior	Target	Result
1	0	2021-11-04	12:30	-3.82507	-40.89322			Alceu	Male	Juvenile 2	Digging	USO	Unsuccessful
1	0	2021-11-04	13:01	-3.82507	-40.89322			Severino	Male	Subadult	Digging	Unknown	Success
1	0	2021-11-12	15:00	-3.82518	-40.89288				Unknown	Juvenile 1	Digging	Unknown	Unsuccessful
1	0	2021-11-15	15:29	-3.82645	-40.89714	SO_UB_281		JoaoGrilo	Male	Subadult	Digging	USO	Unsuccessful
1	0	2021-11-18	08:22	-3.82547	-40.89571			Canjica	Male	Subadult	Digging	USO	Success
1	0	2021-11-18	08:32	-3.82547	-40.89571			JoaoGrilo	Male	Subadult	Digging	USO	Success
1	0	2021-11-22	09:12	-3.82495	-40.89296	SO_UB_739		Lirinha	Male	Juvenile 2	Digging	Unknown	Unsuccessful
1	0	2021-11-22	10:16	-3.82457	-40.89340				Unknown	Juvenile 2	Digging	Unknown	Unsuccessful
1	0	2021-11-22	14:39	-3.82812	-40.89583			Lirinha	Male	Juvenile 2	Digging	Unknown	Unsuccessful
1	0	2021-11-23	09:03	-3.82432	-40.89221				Unknown	Juvenile 2	Digging	Unknown	Success
1	0	2021-12-01	12:22	-3.82464	-40.89256	SO_UB_309		Lirinha	Male	Juvenile 2	Digging	Unknown	Unsuccessful
1	0	2021-12-01	12:36	-3.92448	-40.89263			JoaoGrilo	Male	Subadult	Digging	USO	Unsuccessful
1	0	2021-12-01	12:41	-3.92448	-40.89263				Male	Juvenile 2	Digging	Unknown	Unsuccessful
1	0	2022-03-14	13:29	-3.82463	-40.89247			Baiao	Male	Adult	Digging	USO	Unsuccessful
1	0	2022-03-14	13:37	-3.82463	-40.89247			Mucunza	Male	Subadult	Digging	USO	Success
1	0	2022-03-14	13:47	-3.82432	-40.89237			JoaoGrilo	Male	Subadult	Digging	Unknown	Unsuccessful
1	0	2022-03-14	13:56	-3.82425	-40.89240			JoaoGrilo	Male	Subadult	Digging	USO	Success
1	0	2022-04-21	14:20	-3.82637	-40.89713	SO_UB_741		Mucunza	Male	Subadult	Digging	USO	Success
1	0	2022-06-17	13:30	-3.82662	-40.89762			MariaBonita	Female	Adult	Digging	USO	Unsuccessful
1	0	2022-08-02	10:00	-3.82567	-40.89326			Fabiano	Male	Adult	Digging	USO	Success
1	0	2022-08-02	10:03	-3.82567	-40.89326			Fabiano	Male	Adult	Digging	USO	Success
1	0	2022-08-05	11:22	-3.82486	-40.89610			Canjica	Male	Subadult	Digging	Unknown	Unsuccessful
1	0	2022-08-05	11:32	-3.82486	-40.89610	SO_UB_514		Canjica	Male	Subadult	Digging	USO	Unsuccessful
1	0	2022-08-05	11:34	-3.82486	-40.89610	SO_UB_514	SO_UB_515	Alceu	Male	Juvenile 2	Digging	USO	Unsuccessful
1	0	2022-09-15	14:15	-3.82408	-40.90136			Canjica	Male	Subadult	Digging	USO	Unsuccessful
1	0	2022-09-19	15:10	-3.82663	-40.89783			Chico	Male	Adult	Digging	USO	Unsuccessful
1	0	2022-09-27	12:45	-3.82712	-40.90037			Alceu	Male	Juvenile 2	Digging	Unknown	Unsuccessful
1	0	2022-10-07	13:35	-3.82524	-40.89333	SO_UB_683		Canjica	Male	Subadult	Digging	USO	Unsuccessful
1	0	2022-10-12	15:36	-3.82898	-40.89922			Chico	Male	Adult	Digging	Spider burrow	Unsuccessful
1	0	2022-10-13	11:32	-3.82590	-40.89269			Alceu	Male	Juvenile 2	Digging	Spider burrow	Unsuccessful
1	0	2022-10-15	15:01	-3.82590	-40.89371			Mucunza	Male	Subadult	Digging	Unknown	Success
1	0	2022-10-18	13:40	-3.82518	-40.89306	SO_UB_706		JoaoGrilo	Male	Subadult	Digging	USO	Success
1	0	2022-10-21	12:15	-3.82522	-40.89293			Canjica	Male	Subadult	Digging	USO	Unsuccessful
1	0	2022-10-24	08:37	-3.82638	-40.89492	SO_UB_709		JoaoGrilo	Male	Subadult	Digging	Spider burrow	Success
1	0	2022-10-31	13:40	-3.82610	-40.89290	SO_UB_744		Braulio	Male	Juvenile 2	Digging	Spider burrow	Success
1	0	2022-11-01	10:09	-3.82646	-40.89717	SO_UB_742		Canjica	Male	Subadult	Digging	Unknown	Unsuccessful
1	0	2022-11-01	10:15	-3.82632	-40.89720			JoaoGrilo	Male	Subadult	Digging	USO	Unsuccessful
1	0	2022-11-01	10:35	-3.82650	-40.89788	SO_UB_745		Mucunza	Male	Subadult	Digging	Unknown	Unsuccessful
1	0	2022-11-01	10:36	-3.82647	-40.89790			Mucunza	Male	Subadult	Digging	USO	Unsuccessful
1	0	2022-11-02	15:03	-3.82855	-40.89463	SO_UB_743		Chico	Male	Subadult	Digging	USO	Success
1	0	2022-11-02	15:16	-3.82865	-40.89480			Canjica	Male	Subadult	Digging	Unknown	Unsuccessful
1	0	2022-11-02	15:26	-3.82852	-40.89491	SO_UB_718		Chico	Male	Adult	Digging	Unknown	Unsuccessful
1	0	2022-11-05	09:03	-3.82560	-40.89531			Canjica	Male	Subadult	Digging	Unknown	Unsuccessful
1	0	2022-11-05	09:11	-3.82560	-40.89531			JoaoGrilo	Male	Subadult	Digging	USO	Unsuccessful
1	0	2022-11-05	16:20	-3.82574	-40.89682			Mucunza	Male	Subadult	Digging	Unknown	Unsuccessful
1	0	2022-11-11	08:44	-3.82558	-40.89697	SO_UB_729		Fabiano	Male	Adult	Digging	Spider burrow	Unsuccessful
1	0	2022-11-23	13:23	-3.82757	-40.89918				Unknown	Juvenile 1	Digging	Unknown	Unsuccessful
1	0	2022-11-23	15:56	-3.82660	-40.89749			JoaoGrilo	Male	Subadult	Digging	USO	Unsuccessful
1	0	2022-11-25	10:16	-3.82591	-40.89708	SO_UB_738		Baleia	Female	Adult	Digging	Spider burrow	Success
1	1	2022-10-07	13:13	-3.82514	-40.89290			JoaoGrilo	Male	Subadult	Digging	Spider burrow	Success
1	1	2022-10-21	10:56	-3.82586	-40.89308			JoaoGrilo	Male	Subadult	Digging	Spider burrow	Success
1	0	2022-10-21	11:41	-3.82531	-40.89242			Canjica	Male	Subadult	Digging	Spider burrow	Unsuccessful
1	1	2022-10-21	12:20	-3.82619	-40.89303	SO_UB_719		JoaoGrilo	Male	Subadult	Digging	Spider burrow	Success
1	1	2022-10-31	09:00	-3.82633	-40.89513			Fabiano	Male	Adult	Digging	Spider burrow	Success
1	0	2022-12-06	12:22	-3.82628	-40.89332			Chico	Male	Adult	Digging	Spider burrow	Unsuccessful
1	0	2022-12-06	15:11	-3.82601	-40.89498			Chico	Male	Adult	Digging	Spider burrow	Unsuccessful
1	1	2022-12-08	16:10	-3.82574	-40.89264			Canjica	Male	Subadult	Digging	Spider burrow	Unsuccessful
1	0	2022-12-08	16:11	-3.82470	-40.89309	SO_UB_806		Canjica	Male	Subadult	Digging	Spider burrow	Success
1	0	2022-12-09	15:52	-3.82517	-40.89300	SO_UB_764		JoaoGrilo	Male	Subadult	Digging	USO	Unsuccessful
1	0	2022-12-09	15:53	-3.82515	-40.89311	SO_UB_765		JoaoGrilo	Male	Subadult	Digging	USO	Unsuccessful
1	0	2022-12-11	13:53	-3.82523	-40.89289			JoaoGrilo	Male	Subadult	Digging	USO	Unsuccessful
1	0	2022-12-11	13:54	-3.82526	-40.89298				Male	Juvenile 1	Digging	Unknown	Unsuccessful
1	0	2022-12-11	14:11	-3.82493	-40.89308	SO_UB_807		Canjica	Male	Subadult	Digging	Spider burrow	Success
1	1	2022-12-17	14:06	-3.82472	-40.89203	SO_UB_788		Canjica	Male	Subadult	Digging	Spider burrow	Success
1	0	2023-01-04	13:36	-3.82574	-40.89320			Braulio	Male	Juvenile 2	Digging	Unknown	Unsuccessful
1	0	2023-01-05	15:31	-3.82666	-40.89773	SO_UB_1123		Mucunza	Male	Subadult	Digging	USO	Unsuccessful
1	0	2023-01-06	15:43	-3.82524	-40.89600			Canjica	Male	Subadult	Digging	Unknown	Unsuccessful
1	0	2023-01-06	15:45	-3.82526	-40.89603			Chico	Male	Adult	Digging	Unknown	Unsuccessful
1	0	2023-01-10	9:52	-3.82578	-40.89673			Canjica	Male	Subadult	Digging	USO	Unsuccessful
1	0	2023-01-10	9:57	-3.82569	-40.89650	SO_UB_1124		Canjica	Male	Subadult	Digging	USO	Unsuccessful
1	0	2023-01-12	14:51	-3.82654	-40.89735			JoaoGrilo	Male	Subadult	Digging	USO	Unsuccessful
1	0	2023-01-12	14:53	-3.82652	-40.89746			JoaoGrilo	Male	Subadult	Digging	USO	Unsuccessful
1	0	2023-01-12	14:58	-3.82646	-40.89722	SO_UB_1125A	SO_UB_1125B	Chico	Male	Adult	Digging	USO	Unsuccessful
1	0	2023-01-15	8:11	-3.82522	-40.89574			Canjica	Male	Subadult	Digging	Unknown	Unsuccessful
1	0	2023-01-15	11:35	-3.82587	-40.89651	SO_UB_1126		Canjica	Male	Subadult	Digging	USO	Unsuccessful
1	0	2023-01-15	11:46	-3.82574	-40.89666			Canjica	Male	Subadult	Digging	USO	Unsuccessful
1	0	2023-01-19	15:06	-3.82598	-40.89492	SO_UB_1128			Unknown	Juvenile 1	Digging	USO	Unsuccessful
1	0	2023-01-25	7:39	-3.82541	-40.89548	SO_UB_1129A	SO_UB_1129B	Mucunza	Male	Subadult	Digging	Unknown	Unsuccessful
1	0	2023-03-02	9:11	-3.82576	-40.89321			Canjica	Male	Subadult	Digging	Unknown	Unsuccessful
1	0	2023-05-05	11:25	-3.82818	-40.89444	SO_UB_1131		Canjica	Male	Subadult	Digging	USO	Success
1	0	2023-05-05	12:51	-3.82818	-40.89479	SO_UB_1132		Canjica	Male	Subadult	Digging	USO	Success
1	0	2023-05-08	12:55	-3.82802	-40.89459	SO_UB_1133		Canjica	Male	Subadult	Digging	USO	Unsuccessful
1	0	2023-05-16	14:28	-3.82639	-40.89720	SO_UB_1134		Mucunza	Male	Subadult	Digging	USO	Unsuccessful
1	0	2023-05-17	14:29	-3.82633	-40.89758	SO_UB_1136		Canjica	Male	Subadult	Digging	USO	Unsuccessful
1	0	2023-05-17	14:32	-3.82642	-40.89726	SO_UB_1135A	SO_UB_1135B	JoaoGrilo	Male	Subadult	Digging	USO	Unsuccessful
1	0	2023-05-18	12:43	-3.82812	-40.89454	SO_UB_1137		Mucunza	Male	Subadult	Digging	USO	Unsuccessful
1	0	2023-05-18	12:57	-3.82798	-40.89461	SO_UB_1138		Braulio	Male	Juvenile 2	Digging	USO	Unsuccessful
1	0	2023-05-21	15:29	-3.82667	-40.89751	SO_UB_1139		Alceu	Male	Juvenile 2	Digging	USO	Unsuccessful
1	0	2023-05-21	15:41	-3.82647	-40.89778	SO_UB_1140		Canjica	Male	Subadult	Digging	USO	Unsuccessful
1	0	2023-05-30	14:45	-3.82812	-40.89522	SO_UB_1141A	SO_UB_1141B	Fabiano	Male	Adult	Digging	USO	Unsuccessful

1	0	2023-05-31	15:31	-3.82877	-40.89517			Chico	Male	Adult	Digging	USO	Unsuccessful
1	0	2023-06-02	15:35	-3.82598	-40.89419			Cuscuz	Male	Adult	Digging	USO	Success
1	0	2023-06-03	11:11	-3.82432	-40.89252			Canjica	Male	Subadult	Digging	USO	Success
1	0	2023-06-06	7:52	-3.82565	-40.89498			Mucunza	Male	Subadult	Digging	Spider burrow	Unsuccessful
1	0	2023-06-07	8:03	-3.82561	-40.89347			Canjica	Male	Subadult	Digging	USO	Unsuccessful
1	0	2023-06-13	9:17	-3.82428	-40.89201	SO_UB_1142		Alceu	Male	Juvenile 2	Digging	USO	Success
1	0	2023-06-17	10:48	-3.82577	-40.89122	SO_UB_1143		Mucunza	Male	Subadult	Digging	USO	Success
1	0	2023-06-17	10:50	-3.82580	-40.89113	SO_UB_1144		Fabiano	Male	Adult	Digging	Spider burrow	Success
1	0	2023-07-12	10:21	-3.82576	-40.89596	SO_UB_1145		Mucunza	Male	Subadult	Digging	USO	Unsuccessful
1	0	2023-07-12	10:32	-3.82567	-40.89602	SO_UB_1146		Mucunza	Male	Subadult	Digging	USO	Success
1	0	2023-07-12	13:00	-3.82656	-40.89729			Canjica	Male	Subadult	Digging	Unknown	Unsuccessful
1	0	2023-07-12	13:04	-3.82653	-40.89736			Canjica	Male	Subadult	Digging	Unknown	Unsuccessful
1	0	2023-07-12	14:03	-3.82551	-40.89681			Fabiano	Male	Adult	Digging	USO	Success
1	0	2023-07-12	14:07	-3.82546	-40.89693			MariaBonita	Female	Adult	Digging	USO	Unsuccessful
1	0	2023-07-13	14:54	-3.82613	-40.89470			Mucunza	Male	Subadult	Digging	Unknown	Unsuccessful
1	0	2023-07-29	13:33	-3.82573	-40.89323			Cajuina	Female	Adult	Digging	USO	Success
1	0	2023-07-29	13:34	-3.82573	-40.89323			Fabiano	Male	Adult	Digging	USO	Success
1	0	2023-07-29	13:39	-3.82573	-40.89323			Mucunza	Male	Subadult	Digging	USO	Unsuccessful
1	0	2023-07-29	13:48	-3.82596	-40.89349				Unknown	Juvenile 2	Digging	USO	Success
1	0	2023-07-29	14:04	-3.82578	-40.89340			JoaoGrilo	Male	Subadult	Digging	USO	Success
0	0	2021-10-09	15:05	-3.82649	-40.89471			Mucunza	Male	Subadult	Digging	Unknown	Unsuccessful
0	0	2021-10-22	09:04	-3.82712	-40.89567			Cuscuz	Male	Adult	Digging	Spider burrow	Success
0	0	2021-11-04	12:27	-3.82507	-40.89322			Canjica	Male	Subadult	Digging	USO	Unsuccessful
0	0	2021-11-18	08:14	-3.82547	-40.89571				Male	Juvenile 2	Digging	USO	Unsuccessful
0	0	2021-11-19	09:51	-3.92632	-40.89438			Alceu	Male	Juvenile 2	Digging	Unknown	Success
0	0	2021-11-22	09:53	-3.82460	-40.89337				Unknown	Juvenile 2	Digging	Unknown	Unsuccessful
0	0	2021-12-01	11:38	-3.82582	-40.89261			Baleia	Female	Adult	Digging	Spider burrow	Unsuccessful
0	0	2021-12-01	12:17	-3.82477	-40.89255			Baleia	Female	Adult	Digging	Unknown	Unsuccessful
0	0	2022-02-21	12:42	-3.82484	-40.89297			Alceu	Male	Juvenile 2	Digging	Unknown	Unsuccessful
0	0	2022-02-25	12:42	-3.82633	-40.89582			Mucunza	Male	Subadult	Digging	Unknown	Unsuccessful
0	0	2022-03-01	15:18	-3.82746	-40.89752			Severino	Male	Subadult	Digging	Unknown	Unsuccessful
0	0	2022-03-14	13:28	-3.82463	-40.89247			Cordel	Female	Adult	Digging	USO	Unsuccessful
0	0	2022-03-14	13:31	-3.82463	-40.89247			Mucunza	Male	Subadult	Digging	USO	Unsuccessful
0	0	2022-04-08	14:27	-3.82587	-40.89775			JoaoGrilo	Male	Subadult	Digging	USO	Unsuccessful
0	0	2022-04-08	14:36	-3.82587	-40.89782			Fabiano	Male	Adult	Digging	USO	Success
0	0	2022-04-15	13:52	-3.82577	-40.89405			JoaoGrilo	Male	Adult	Digging	USO	Success
0	0	2022-04-21	14:26	-3.82639	-40.89708			Braulio	Male	Juvenile 2	Digging	USO	Success
0	0	2022-05-17	13:37	-3.82678	-40.89756			Lirinha	Male	Juvenile 2	Digging	Unknown	Unsuccessful
0	0	2022-05-17	13:38	-3.82678	-40.89756			Braulio	Male	Juvenile 2	Digging	Unknown	Success
0	0	2022-05-17	13:55	-3.82678	-40.89756			Braulio	Male	Juvenile 2	Digging	USO	Success
0	0	2022-06-17	13:09	-3.82636	-40.89702			Baleia	Female	Adult	Digging	USO	Success
0	0	2022-06-17	13:28	-3.82662	-40.89762				Female	Juvenile 2	Digging	USO	Unsuccessful
0	0	2022-06-21	12:15	-3.82511	-40.89093			Chico	Male	Adult	Digging	Spider burrow	Success
0	0	2022-06-30	14:21	-3.82548	-40.89097			Maria Moura	Female	Adult	Digging	Spider burrow	Success
0	0	2022-08-02	08:52	-3.82617	-40.89496			Fabiano	Male	Adult	Digging	USO	Success
0	0	2022-08-02	09:05	-3.82607	-40.89456			Fabiano	Male	Adult	Digging	USO	Success
0	0	2022-08-02	09:53	-3.82576	-40.89339			Cordel	Female	Adult	Digging	USO	Success
0	0	2022-08-05	11:21	-3.82504	-40.89603			Mucunza	Male	Subadult	Digging	Unknown	Unsuccessful
0	0	2022-09-19	15:07	-3.82656	-40.89786			Chico	Male	Adult	Digging	USO	Unsuccessful
0	0	2022-09-30	11:52	-3.82616	-40.89231			JoaoGrilo	Male	Subadult	Digging	Unknown	Unsuccessful
0	0	2022-10-07	13:11	-3.82514	-40.89290			Baleia	Female	Adult	Digging	USO	Success
0	0	2022-10-12	13:44	-3.82811	-40.89812			Conceicao	Female	Adult	Digging	Spider burrow	Unsuccessful
0	0	2022-10-12	14:07	-3.82811	-40.89812			Chico	Male	Adult	Digging	Spider burrow	Success
0	0	2022-10-12	14:27	-3.82811	-40.89812			Conceicao	Female	Adult	Digging	Unknown	Unsuccessful
0	0	2022-10-12	14:29	-3.82811	-40.89812			Conceicao	Female	Adult	Digging	Unknown	Unsuccessful
0	0	2022-10-12	14:31	-3.82812	-40.89821			Conceicao	Female	Adult	Digging	Spider burrow	Success
0	0	2022-10-12	14:32	-3.82812	-40.89821			Conceicao	Female	Adult	Digging	Spider burrow	Unsuccessful
0	0	2022-10-12	15:36	-3.82904	-40.89930			Conceicao	Female	Adult	Digging	Spider burrow	Success
0	0	2022-10-13	11:27	-3.82586	-40.89266				Unknown	Juvenile 2	Digging	Unknown	Unsuccessful
0	0	2022-10-13	11:40	-3.82586	-40.89266				Female	Juvenile 1	Digging	Unknown	Unsuccessful
0	0	2022-10-15	13:35	-3.82654	-40.89351			JoaoGrilo	Male	Subadult	Digging	Unknown	Unsuccessful
0	0	2022-10-15	15:31	-3.82590	-40.89371			Chico	Male	Adult	Digging	USO	Success
0	0	2022-11-01	09:54	-3.82649	-40.89647			Conceicao	Female	Adult	Digging	Unknown	Unsuccessful
0	0	2022-11-01	10:32	-3.82650	-40.89788			Mucunza	Male	Subadult	Digging	USO	Unsuccessful
0	0	2022-11-02	14:36	-3.82822	-40.89450			JoaoGrilo	Male	Subadult	Digging	USO	Unsuccessful
0	0	2022-11-05	07:20	-3.82658	-40.89695			Canjica	Male	Subadult	Digging	Unknown	Unsuccessful
0	0	2022-11-05	08:50	-3.82562	-40.89531			JoaoGrilo	Male	Subadult	Digging	Unknown	Unsuccessful
0	0	2022-11-05	08:55	-3.82562	-40.89531			JoaoGrilo	Male	Subadult	Digging	Unknown	Unsuccessful
0	0	2022-11-05	09:00	-3.82562	-40.89531			Alceu	Male	Juvenile 2	Digging	Unknown	Unsuccessful
0	0	2022-11-05	09:13	-3.28538	-40.89504			Mucunza	Male	Subadult	Digging	Unknown	Success
0	0	2022-11-05	09:47	-3.82597	-40.89468			Mucunza	Male	Subadult	Digging	Unknown	Unsuccessful
0	0	2022-11-14	14:56	-3.82639	-40.89794			Fabiano	Male	Adult	Digging	Unknown	Unsuccessful
0	0	2022-11-20	10:36	-3.82726	-40.89760			Acucena	Female	Adult	Digging	Unknown	Unsuccessful
0	0	2022-11-21	12:50	-3.82815	-40.89841			Conceicao	Female	Adult	Digging	Spider burrow	Success
0	0	2022-11-23	15:14	-3.82741	-40.89822			Fabiano	Male	Adult	Digging	Unknown	Unsuccessful
0	0	2022-11-25	09:56	-3.82564	-40.89731			MariaBonita	Female	Adult	Digging	USO	Unsuccessful
0	0	2022-11-26	15:32	-3.82561	-40.89668			Cordel	Female	Adult	Digging	Unknown	Success
0	0	2022-12-08	13:04	-3.82742	-40.89553			Xote	Female	Adult	Digging	Unknown	Unsuccessful
0	0	2022-12-17	7:31	-3.82555	-40.89588			Xote	Female	Adult	Digging	Unknown	Unsuccessful
0	0	2022-12-17	13:59	-3.82473	-40.89199			Tapioca	Female	Adult	Digging	Spider burrow	Unsuccessful
0	0	2022-12-17	13:59	-3.82476	-40.89200			Baleia	Female	Adult	Digging	Spider burrow	Success
0	0	2022-12-18	15:11	-3.82348	-40.89232			Acucena	Female	Adult	Digging	Spider burrow	Success
0	0	2023-01-04	9:11	-3.82576	-40.89498			Cajuina	Female	Adult	Digging	Spider burrow	Success
0	0	2023-01-04	13:37	-3.82574	-40.89320			Canjica	Male	Subadult	Digging	Unknown	Unsuccessful
0	0	2023-01-04	13:36	-3.82574	-40.89320			Cajuina	Female	Adult	Digging	Spider burrow	Unsuccessful
0	0	2023-01-05	14:28	-3.82583	-40.89673			Canjica	Male	Subadult	Digging	USO	Unsuccessful
0	0	2023-01-06	15:10	-3.82564	-40.89606			Chico	Male	Adult	Digging	USO	Unsuccessful
0	0	2023-01-06	15:48	-3.82527	-40.89616			JoaoGrilo	Male	Adult	Digging	Spider burrow	Success
0	0	2023-01-13	15:36	-3.82668	-40.89573			MariaBonita	Female	Adult	Digging	Unknown	Success
0	0	2023-01-19	9:13	-3.82621	-40.89598			Chico	Male	Adult	Digging	Unknown	Success
0	0	2023-01-19	9:15	-3.82620	-40.89604			Acucena	Female	Adult	Digging	Unknown	Unsuccessful
0	0	2023-01-19	9:16	-3.82620	-40.89604			Chico	Male	Adult	Digging	Unknown	Unsuccessful
0	0	2023-01-19	15:02	-3.82598	-40.89493			Mucunza	Male	Subadult	Digging	USO	Success

0	0	2023-01-19	15:05	-3.82598	-40.89493			Mucunza	Male	Subadult	Digging	USO	Unsuccessful
0	0	2023-02-20	9:37	-3.82549	-40.89394			Conceicao	Female	Adult	Digging	USO	Unsuccessful
0	0	2023-03-02	11:30	-3.82713	-40.89806			Chico	Male	Adult	Digging	USO	Success
0	0	2023-03-10	9:51	-3.82465	-40.89245			Cajuina	Female	Adult	Digging	USO	Success
0	0	2023-04-04	11:59	-3.82752	-40.89227			JoaoGrilo	Male	Subadult	Digging	USO	Success
0	0	2023-04-04	14:10	-3.82644	-40.89706			Maria Moura	Female	Adult	Digging	USO	Success
0	0	2023-04-04	14:29	-3.82664	-40.89763			Mucunza	Male	Subadult	Digging	Unknown	Unsuccessful
0	0	2023-04-08	9:28	-3.82655	-40.89170			JoaoGrilo	Male	Subadult	Digging	Unknown	Success
0	0	2023-04-12	12:29	-3.82653	-40.89735			Baleia	Female	Adult	Digging	USO	Success
0	0	2023-04-24	13:06	-3.82545	-40.89167			Braulio	Male	Juvenile 2	Digging	USO	Unsuccessful
0	0	2023-04-28	12:16	-3.82476	-40.89250			Canjica	Male	Subadult	Digging	USO	Success
0	0	2023-05-05	11:25	-3.82818	-40.89444			Canjica	Male	Subadult	Digging	USO	Success
0	0	2023-05-07	14:30	-3.82630	-40.89721			Tapioca	Female	Adult	Digging	USO	Unsuccessful
0	0	2023-05-18	11:55	-3.82820	-40.89388				Unknown	Juvenile 1	Digging	Unknown	Unsuccessful
0	0	2023-05-18	12:41	-3.82814	-40.89458			Mucunza	Male	Subadult	Digging	Unknown	Unsuccessful
0	0	2023-05-18	12:55	-3.82800	-40.89456			Braulio	Male	Juvenile 2	Digging	USO	Unsuccessful
0	0	2023-05-21	15:29	-3.82672	-40.89754			Arapua	Female	Juvenile 2	Digging	Unknown	Unsuccessful
0	0	2023-06-03	11:17	-3.82461	-40.89241			Chico	Male	Adult	Digging	USO	Success
0	0	2023-06-06	7:53	-3.82565	-40.89498			Canjica	Male	Subadult	Digging	Spider burrow	Unsuccessful
0	0	2023-06-06	7:54	-3.82565	-40.89498			Braulio	Male	Juvenile 2	Digging	Spider burrow	Unsuccessful
0	0	2023-06-06	11:40	-3.82594	-40.89125			Fabiano	Male	Adult	Digging	Spider burrow	Success
0	0	2023-06-17	10:38	-3.82586	-40.89120			Maria Moura	Female	Adult	Digging	Spider burrow	Success
0	0	2023-06-17	10:42	-3.82586	-40.89120			Maria Moura	Female	Adult	Digging	Unknown	Success
0	0	2023-07-05	14:21	-3.82638	-40.89297			Maria Moura	Female	Adult	Digging	Spider burrow	Success
0	0	2023-07-12	10:12	-3.82572	-40.89589			Maria Moura	Female	Adult	Digging	USO	Unsuccessful
0	0	2023-07-13	14:55	-3.82604	-40.89485			Mucunza	Male	Subadult	Digging	USO	Unsuccessful
0	0	2023-07-13	14:57	-3.82613	-40.89470			Lirinha	Male	Juvenile 2	Digging	USO	Unsuccessful
0	0	2023-07-14	7:57	-3.82602	-40.89650			Betania	Female	Adult	Digging	USO	Success
0	0	2023-07-29	8:44	-3.82619	-40.89501			JoaoGrilo	Male	Subadult	Digging	USO	Unsuccessful
0	0	2023-07-29	13:33	-3.82573	-40.89323			Maria Moura	Female	Adult	Digging	USO	Unsuccessful
0	0	2023-07-29	14:12	-3.82578	-40.89340			Braulio	Male	Juvenile 2	Digging	USO	Unsuccessful

Stone tools

Tool	Date	Time	Place	Lat	Long	Length (cm)	Width (cm)	Tickness (cm)	Weigth (g)	Material	Target	Group	Result	Individual
SO_UB_281	2021-11-15	15:29	UNP	-3.82645	-40.89714	8.6	3.4	3.3	80.2	Sandstone	USO	Sertão	Unsuccessful	JoaoGrilo
SO_UB_739	2021-11-22	09:12	UNP	-3.82495	-40.89296	6.0	3.0	2.2	60.3	Sandstone	Unknown	Sertão	Unsuccessful	Lirinha
SO_UB_309	2021-12-01	12:22	UNP	-3.82464	-40.89256	8.2	3.3	2.4	73.8	Sandstone	Unknown	Sertão	Unsuccessful	Lirinha
SO_UB_741	2022-04-21	14:20	UNP	-3.82637	-40.89713	9.0	5.0	3.4		Sandstone	USO	Sertão	Success	Mucunza
SO_UB_514	2022-08-05	11:32	UNP	-3.82486	-40.89610	7.9	3.4	3.0	117.4	Sandstone	USO	Sertão	Unsuccessful	Canjica
SO_UB_515	2022-08-05	11:34	UNP	-3.82486	-40.89610	6.3	4.5	3.9	94.0	Sandstone	USO	Sertão	Unsuccessful	Alceu
SO_UB_683	2022-10-07	13:35	UNP	-3.82524	-40.89333	5.4	4.6	2.4	64.8	Sandstone	USO	Sertão	Unsuccessful	Canjica
SO_UB_706	2022-10-18	13:40	UNP	-3.82518	-40.89306	5.7	4.5	3.4	64.3	Sandstone	USO	Sertão	Success	JoaoGrilo
SO_UB_719	2022-10-21	12:20	UNP	-3.82619	-40.89303	10.8	5.4	3.9	202.8	Sandstone	Spider burrow	Sertão	Success	JoaoGrilo
SO_UB_709	2022-10-24	08:37	UNP	-3.82638	-40.89492	8.1	4.5	1.7	69.3	Sandstone	Spider burrow	Sertão	Success	JoaoGrilo
SO_UB_718	2022-11-02	15:26	UNP	-3.82852	-40.89491	6.8	5.0	2.9	96.6	Limonite	Unknown	Sertão	Unsuccessful	Chico
SO_UB_729	2022-11-11	08:44	UNP	-3.82558	-40.89697	5.7	4.9	3.0	58.4	Sandstone	Spider burrow	Sertão	Unsuccessful	Fabiano
SO_UB_738	2022-11-25	10:16	UNP	-3.82591	-40.89708	5.1	3.0	2.4	39.2	Sandstone	Spider burrow	Sertão	Success	Baleia
SO_UB_742	2022-11-01	10:09	UNP	-3.82646	-40.89717	5.9	5.0	3.2	87.1	Sandstone	Unknown	Sertão	Unsuccessful	Canjica
SO_UB_743	2022-11-02	15:03	UNP	-3.82855	-40.89463	9.4	6.5	2.8	168.4	Sandstone	USO	Sertão	Success	Chico
SO_UB_744	2022-10-31	13:40	UNP	-3.82610	-40.89290	5.0	4.2	3.4	67.4	Sandstone	Spider burrow	Sertão	Success	Braulio
SO_UB_745	2022-11-01	10:35	UNP	-3.82650	-40.89788	8.7	6.5	2.3	103.6	Sandstone	Unknown	Sertão	Unsuccessful	Mucunza
SO_UB_806	2022-12-08	16:11	UNP	-3.82470	-40.89309	7.0	4.0	3.2	113.5	Sandstone	Spider burrow	Sertão	Success	Canjica
SO_UB_764	2022-12-09	15:52	UNP	-3.82515	-40.89311	6.7	4.8	1.5	52.0	Sandstone	USO	Sertão	Unsuccessful	JoaoGrilo
SO_UB_765	2022-12-09	15:53	UNP	-3.82517	-40.89300	6.4	5.3	4.1	115.0	Sandstone	USO	Sertão	Unsuccessful	JoaoGrilo
SO_UB_807	2022-12-11	14:11	UNP	-3.82493	-40.89308	8.3	7.1	2.6	134.5	Sandstone	Spider burrow	Sertão	Success	Canjica
SO_UB_788	2022-12-17	14:08	UNP	-3.82472	-40.89203	5.6	3.7	3.7	96.4	Sandstone	Spider burrow	Sertão	Success	Canjica
SO_UB_1123	2023-01-05	15:31	UNP	-3.82666	-40.89773	9.6	6.2	3.1	152.5	Sandstone	USO	Sertão	Unsuccessful	Mucunza
SO_UB_1124	2023-01-10	9:57	UNP	-3.82569	-40.89650	7.4	5.0	1.8	69.4	Sandstone	USO	Sertão	Unsuccessful	Canjica
SO_UB_1125A	2023-01-12	14:58	UNP	-3.82646	-40.89722	11.1	6.4	2.7	176.8	Sandstone	USO	Sertão	Unsuccessful	Chico
SO_UB_1125B	2023-01-12	14:58	UNP	-3.82646	-40.89722	6.9	6.1	3.8	142.0	Sandstone	USO	Sertão	Unsuccessful	Chico
SO_UB_1126	2023-01-15	11:35	UNP	-3.82587	-40.89651	7.8	5.0	2.0	87.6	Sandstone	USO	Sertão	Unsuccessful	Canjica
SO_UB_1127	2023-01-19	9:58	UNP	-3.82579	-40.89676	4.6	3.6	2.4	27.2	Sandstone	USO	Sertão	Unsuccessful	Canjica
SO_UB_1128	2023-01-19	15:06	UNP	-3.82598	-40.89492	7.3	6.2	2.3	113.3	Sandstone	USO	Sertão	Unsuccessful	Unknown
SO_UB_1129A	2023-01-25	7:39	UNP	-3.82541	-40.89548	8.5	4.5	1.9	77.3	Sandstone	Unknown	Sertão	Unsuccessful	Mucunza
SO_UB_1129B	2023-01-25	7:39	UNP	-3.82541	-40.89548	13.6	10.2	3.0	411.7	Sandstone	Unknown	Sertão	Unsuccessful	Mucunza
SO_UB_1130	2023-03-02	11:35	UNP	-3.82705	-40.89807	6.4	4.0	2.5	59.6	Sandstone	Unknown	Sertão	Unsuccessful	Chico
SO_UB_1131	2023-05-05	11:25	UNP	-3.82818	-40.89444	6.5	6.3	4.2	131.5	Sandstone	USO	Sertão	Unsuccessful	Canjica
SO_UB_1132	2023-05-05	12:51	UNP	-3.82818	-40.89479	11.5	5.0	3.5	156.9	Sandstone	USO	Sertão	Success	Canjica
SO_UB_1133	2023-05-08	12:55	UNP	-3.82802	-40.89459	7.1	5.5	2.0	71.4	Sandstone	USO	Sertão	Unsuccessful	Canjica
SO_UB_1134	2023-05-16	14:28	UNP	-3.82639	-40.89720	8.2	5.5	2.9	113.0	Sandstone	USO	Sertão	Unsuccessful	Mucunza
SO_UB_1135A	2023-05-17	14:32	UNP	-3.82642	-40.89726	5.5	4.2	2.9	48.3	Sandstone	USO	Sertão	Unsuccessful	JoaoGrilo
SO_UB_1135B	2023-05-17	14:32	UNP	-3.82642	-40.89726	6.7	6.2	3.3	110.2	Sandstone	USO	Sertão	Unsuccessful	JoaoGrilo
SO_UB_1136	2023-05-17	14:29	UNP	-3.82633	-40.89758	6.8	2.6	1.7	47.9	Sandstone	USO	Sertão	Unsuccessful	Canjica
SO_UB_1137	2023-05-18	12:43	UNP	-3.82812	-40.89454	6.4	5.4	2.6	88.3	Sandstone	USO	Sertão	Unsuccessful	Mucunza
SO_UB_1138	2023-05-18	12:57	UNP	-3.82798	-40.89461	5.1	3.8	2.0	31.3	Sandstone	USO	Sertão	Unsuccessful	Braulio
SO_UB_1139	2023-05-21	15:29	UNP	-3.82667	-40.89751	9.2	8.3	3.5	161.2	Sandstone	USO	Sertão	Unsuccessful	Alceu
SO_UB_1140	2023-05-21	15:41	UNP	-3.82647	-40.89778	6.7	4.3	3.7	95.4	Quartz	USO	Sertão	Unsuccessful	Canjica
SO_UB_1141A	2023-05-30	14:45	UNP	-3.82812	-40.89522	21.3	8.5	3.4	721.9	Sandstone	USO	Sertão	Unsuccessful	Fabiano
SO_UB_1141B	2023-05-30	14:45	UNP	-3.82812	-40.89522	10.5	7.6	4.0	292.9	Limonite	USO	Sertão	Unsuccessful	Fabiano
SO_UB_1142	2023-06-13	9:17	UNP	-3.82428	-40.89201	5.6	4.2	3.2	47.1	Sandstone	USO	Sertão	Success	Alceu
SO_UB_1143	2023-06-17	10:48	UNP	-3.82577	-40.89122	7.4	5.9	3.4	120.7	Quartz	USO	Sertão	Success	Mucunza
SO_UB_1144	2023-06-17	10:50	UNP	-3.82580	-40.89113	8.4	3.2	2.2	102.6	Quartz	Spider burrow	Sertão	Success	Fabiano
SO_UB_1145	2023-07-12	10:21	UNP	-3.82576	-40.89596	11.6	6.0	3.2	326.4	Sandstone	USO	Sertão	Unsuccessful	Mucunza
SO_UB_1146	2023-07-12	10:32	UNP	-3.82567	-40.89602	9.2	6.4	5.3	346.5	Sandstone	USO	Sertão	Success	Mucunza

Probing data

Stone	Season	Date	Time	Individual	Sex	Age	Group	Area	Place	Tool	Lat	Long	Target	Success
0	dry	2021-11-12	13:32	Cuscuz	Male	Adult	Sertão	Vale	UNP	SI_UB_001A, SI_UB_001B	-3.82503	-40.89081	Spider hole	1
0	dry	2021-11-12	14:56	Severino	Male	Subadult	Sertão	Vale	UNP		-3.82515	-40.89291	Ground	0
0	dry	2022-09-30	14:09	João Grilo	Male	Subadult	Sertão	Vale	UNP	SI_UB_002	-3.82631	-40.89333	Spider hole	1
1	dry	2022-10-07	13:13	João Grilo	Male	Subadult	Sertão	Vale	UNP		-3.82514	-40.8929	Spider hole	1
0	dry	2022-10-11	14:19	Fabiano	Male	Adult	Sertão	Vale	UNP		-3.82653	-40.90122	Spider hole	1
0	dry	2022-10-11	14:20	Fabiano	Male	Adult	Sertão	Vale	UNP		-3.82649	-40.90132	Spider hole	1
0	dry	2022-10-12	12:13	Canjica	Male	Subadult	Sertão	Vale	UNP	SI_UB_018	-3.82746	-40.89669	Spider hole	0
0	dry	2022-10-12	14:03	Cuscuz	Male	Adult	Sertão	Vale	UNP	SI_UB_017	-3.82814	-40.89805	Spider hole	1
0	dry	2022-10-13	11:35	João Grilo	Male	Subadult	Sertão	Vale	UNP	SI_UB_015	-3.82589	-40.89261	Ground	1
0	dry	2022-10-13	11:36	João Grilo	Male	Subadult	Sertão	Vale	UNP		-3.82599	-40.89227	Spider hole	0
0	dry	2022-10-13	11:46	João Grilo	Male	Subadult	Sertão	Vale	UNP		-3.8259	-40.89255	Ground	0
0	dry	2022-10-13	11:55	Canjica	Male	Subadult	Sertão	Vale	UNP		-3.8259	-40.89269	Spider hole	1
0	dry	2022-10-13	11:58	Mucunzá	Male	Subadult	Sertão	Vale	UNP	SI_UB_016	-3.8259	-40.89269	Spider hole	0
0	dry	2022-10-13	12:02	João Grilo	Male	Subadult	Sertão	Vale	UNP		-3.82582	-40.89261	Ground	0
0	dry	2022-10-18	10:21	Fabiano	Male	Adult	Sertão	Vale	UNP	SI_UB_004	-3.82635	-40.8946	Ground	0
0	dry	2022-10-18	10:24	Fabiano	Male	Adult	Sertão	Vale	UNP	SI_UB_005A, SI_UB_005B	-3.82635	-40.8946	Spider hole	1
0	dry	2022-10-18	12:25	João Grilo	Male	Subadult	Sertão	Vale	UNP	SI_UB_014	-3.82558	-40.89198	Spider hole	0
0	dry	2022-10-18	13:17	Fabiano	Male	Adult	Sertão	Vale	UNP	SI_UB_013	-3.82511	-40.89257	Spider hole	0
1	dry	2022-10-21	10:56	João Grilo	Male	Subadult	Sertão	Vale	UNP	SI_UB_010	-3.82586	-40.89308	Spider hole	1
0	dry	2022-10-21	11:39	Fabiano	Male	adult	Sertão	Vale	UNP	SI_UB_009	-3.82532	-40.89244	Spider hole	0
0	dry	2022-10-21	11:41	Canjica	Male	Subadult	Sertão	Vale	UNP		-3.82531	-40.89242	Spider hole	1
1	dry	2022-10-21	12:20	João Grilo	Male	Subadult	Sertão	Vale	UNP	SI_UB_011	-3.82619	-40.89303	Spider hole	1
0	dry	2022-10-21	13:11	João Grilo	Male	Subadult	Sertão	Vale	UNP	SI_UB_012	-3.82642	-40.8933	Spider hole	0
1	dry	2022-10-31	9:00	Fabiano	Male	Adult	Sertão	Vale	UNP	SI_UB_021	-3.82633	-40.89513	Spider hole	1
0	dry	2022-10-31	13:06	Fabiano	Male	Adult	Sertão	Vale	UNP	SI_UB_019A, SI_UB_019B	-3.82619	-40.89325	Spider hole	0
0	dry	2022-11-02	9:55	Canjica	Male	Subadult	Sertão	Vale	UNP	SI_UB_007	-3.82705	-40.89647	Spider hole	0
0	dry	2022-11-12	13:17	Fabiano	Male	Adult	Sertão	Vale	UNP		-3.82617	-40.89929	Ground	0
0	dry	2022-11-12	13:36	Canjica	Male	Subadult	Sertão	Vale	UNP		-3.82598	-40.89931	Spider hole	1
0	dry	2022-11-12	13:37	Canjica	Male	Subadult	Sertão	Vale	UNP		-3.82608	-40.89906	Spider hole	0
0	dry	2022-11-13	14:30	Mucunzá	Male	Subadult	Sertão	Vale	UNP		-3.82735	-40.89697	Spider hole	0
0	dry	2022-11-13	14:31	João Grilo	Male	Subadult	Sertão	Vale	UNP	SI_UB_020	-3.82728	-40.89707	Spider hole	0
0	dry	2022-11-16	10:02	João Grilo	Male	Subadult	Sertão	Vale	UNP	SI_UB_023	-3.82667	-40.89766	Spider hole	0
0	dry	2022-11-20	10:38	Fabiano	Male	Adult	Sertão	Vale	UNP		-3.82716	-40.8977	Spider hole	0
0	dry	2022-11-23	13:05	Fabiano	Male	Adult	Sertão	Vale	UNP	SI_UB_024A, SI_UB_024B, SI_UB_024C	-3.82758	-40.89925	Spider hole	1
1	dry	2022-12-08	16:10	Canjica	Male	Subadult	Sertão	Vale	UNP	SI_UB_032	-3.82574	-40.89264	Spider hole	0
0	dry	2022-12-11	14:05	João Grilo	Male	Subadult	Sertão	Vale	UNP		-3.82506	-40.89305	Spider hole	1
0	dry	2022-12-11	14:06	João Grilo	Male	Subadult	Sertão	Vale	UNP	SI_UB_034	-3.82492	-40.89309	Spider hole	0
0	dry	2022-12-12	7:56	Canjica	Male	Subadult	Sertão	Vale	UNP		-3.82593	-40.89458	Ground	0
1	dry	2022-12-17	14:06	Canjica	Male	Subadult	Sertão	Vale	UNP	SI_UB_037	-3.82472	-40.89203	Spider hole	1
0	wet	2023-01-14	11:49	Canjica	Male	Subadult	Sertão	Vale	UNP	SI_UB_040A, SI_UB_040B	-3.8259683	-40.8967667	Ground	0

Stick tools								
**Tool**	**Date**	**Time**	**Place**	**Lat**	**Long**	**Total length (cm)**	**Tickeness (cm)**	**Target**
SI_UB_001A	2021-11-12	13:32	UNP	-3.82503	-40.89081	21	0.2	Spider hole
SI_UB_001B	2021-11-12	13:32	UNP	-3.82503	-40.89081	42	0.2	Spider hole
SI_UB_002	2022-09-30	14:09	UNP	-3.82631	-40.89333	39.1	0.3	Spider hole
SI_UB_004	2022-10-18	10:21	UNP	-3.82635	-40.89460	48.9	0.2	Ground
SI_UB_005A	2022-10-18	10:24	UNP	-3.82635	-40.89460	61.3	0.3	Spider hole
SI_UB_005B	2022-10-18	10:24	UNP	-3.82635	-40.89460	25.5	0.3	Spider hole
SI_UB_007	2022-11-02	9:55	UNP	-3.82705	-40.89647	34.4	0.3	Spider hole
SI_UB_009	2022-10-21	11:39	UNP	-3.82532	-40.89244	41.4	0.4	Spider hole
SI_UB_010	2022-10-21	10:56	UNP	-3.82586	-40.89308	43.9	0.5	Spider hole
SI_UB_011	2022-10-21	12:20	UNP	-3.82619	-40.89303	28.3	0.2	Spider hole
SI_UB_012	2022-10-21	13:11	UNP	-3.82642	-40.89330	26.9	0.7	Spider hole
SI_UB_013	2022-10-18	13:17	UNP	-3.82511	-40.89257	31	0.3	Spider hole
SI_UB_014	2022-10-18	12:25	UNP	-3.82558	-40.89198	27.6	0.3	Spider hole
SI_UB_015	2022-10-13	11:35	UNP	-3.82589	-40.89261	19.5	0.5	Ground
SI_UB_016	2022-10-13	11:58	UNP	-3.82590	-40.89269	8.5	0.5	Spider hole
SI_UB_017	2022-10-12	14:03	UNP	-3.82814	-40.89805	37.5	0.3	Spider hole
SI_UB_018	2022-10-12	12:13	UNP	-3.82746	-40.89669	18.4	0.2	Spider hole
SI_UB_019A	2022-10-31	13:06	UNP	-3.82619	-40.89325	23.8	0.2	Spider hole
SI_UB_019B	2022-10-31	13:06	UNP	-3.82619	-40.89325	45.3	0.25	Spider hole
SI_UB_020	2022-11-13	14:31	UNP	-3.82728	-40.89707	15	0.25	Spider hole
SI_UB_021	2022-10-31	9:00	UNP	-3.82633	-40.89513	26.6	0.3	Spider hole
SI_UB_023	2022-11-16	10:02	UNP	-3.82667	-40.89766	16.7	0.3	Spider hole
SI_UB_024A	2022-11-23	13:05	UNP	-3.82758	-40.89925	21.8	0.2	Spider hole
SI_UB_024B	2022-11-23	13:05	UNP	-3.82758	-40.89925	21.5	0.25	Spider hole
SI_UB_024C	2022-11-23	13:05	UNP	-3.82758	-40.89925	25.1	0.2	Spider hole
SI_UB_032	2022-12-08	16:10	UNP	-3.82574	-40.89264	13.9	0.15	Spider hole
SI_UB_034	2022-12-11	14:06	UNP	-3.82492	-40.89309	48.8	0.2	Spider hole
SI_UB_037	2022-12-17	14:06	UNP	-3.82472	-40.89203	31.8	0.25	Spider hole
SI_UB_040A	2023-01-14	11:49	UNP	-3.82596	-40.89676	23.5	0.25	Ground
SI_UB_040B	2023-01-14	11:49	UNP	-3.82596	-40.89676	12.6	0.2	Ground

Penetrometer data												
**Site**	**Plot**	**Measure 1**	**Measure 2**	**Measure 3**	**Measure 4**	**Measure 5**	**Measure 6**	**Measure 7**	**Measure 8**	**Measure 9**	**Measure 10**	**Mean**
SCaNP	1A	7	6.5	7	7	6.5	7	7.5	7	7	7	6.95
SCaNP	4A	5.5	5	5	4.5	5	4.5	5	4.5	5	6	5
SCaNP	3A	5	6	5	4.5	5	4.5	4.5	5	4.5	5.5	4.95
SCaNP	5A	5.5	5.5	7	6	6	6	6.5	6	6.5	6	6.1
SCaNP	2A	5	4.5	5	5	5	6	5	5.5	5	4.5	5.05
SCaNP	6A	5.5	5.5	5	5.5	6	5	5.5	5.5	6	5.5	5.5
SCaNP	10A	6.5	7	7.5	7	7	6.5	7	7.5	7	6	6.9
SCaNP	9A	7	6	6	6	8	8	5.5	5.5	7.5	6	6.55
SCaNP	8B	6	6.5	6	6	6.5	7	6	6.5	7	6	6.35
SCaNP	7A	4.5	4.5	4.5	6	5.5	5	5.5	6	6.5	4.5	5.25
SCaNP	8A	5	4.5	5.5	5	5	5	6	4.5	4.5	4	4.9
SCaNP	12A	6	5.5	5.5	5	6	5.5	5.5	6	5.5	5	5.55
SCaNP	11B	5.5	3.5	5.5	4	3.5	4	4.5	4.5	6.5	6.5	4.8
SCaNP	12B	6	6.5	6	6	6.5	7	6.5	6.5	6	6.5	6.35
SCaNP	10C	6	6.5	6.5	7	7	7	7	6	6	7	6.6
SCaNP	10B	8	7.5	8.5	7.5	8.5	7	7.5	8	7	7.5	7.7
SCaNP	11A	6.5	6	6.5	5	7	6.5	6.5	5	6	6	6.1
SCaNP	11C	6	6	6	6	6	5	5	6	6	6.5	5.85
SCaNP	10D	6.5	5.5	6	7	7	6	7	6.5	6.5	6	6.4
SCaNP	10E	7	9	8	6	6	6	7.5	7	6.5	7.5	7.05
SCaNP	12D	7.5	8	7.5	7.5	7.5	8	8	7	7.5	7.5	7.6
SCaNP	10F	6	6	5	5.5	4.5	5	7	6.5	5	5.5	5.6
SCaNP	12C	7	7.5	7.5	7.5	6.5	7	7	6.5	6.5	6	6.9
SCaNP	12E	6	6.5	7	7.5	7	5.5	6.5	7	7	6.5	6.65
SCaNP	11D	4.5	4	4.5	4	4	4	3.5	5	5	4.5	4.3
SCaNP	13A	8	9.5	9	8.5	8.5	8	7.5	8	7	8	8.2
SCaNP	13B	6.5	6.5	6.5	7	7	6	6	6	6.5	6	6.4
SCaNP	13C	5	5	5	5.5	5.5	5.5	4.5	5.5	6	5	5.25
SCaNP	16B	8	8	9.5	7.5	7.5	7.5	8	8	7.5	8	7.95
SCaNP	15A	6.5	7	8	8	8.5	8	7.5	7.5	7	8	7.6
SCaNP	14A	6.5	5.5	5.5	6	6	6	6	5.5	6	6	5.9
SCaNP	17C	7.5	8	8	8	8.5	7	8.5	8	7.5	7	7.8
SCaNP	17D	5.5	5	5.5	6	5.5	6.5	6	5.5	6	6	5.75
SCaNP	16C	6	5.5	8	6	5.5	5.5	5.5	5.5	5	4	5.65
SCaNP	16A	6	6.5	5	5	5	5	5.5	5	6.5	5.5	5.5
SCaNP	17B	5.5	6	7	5.5	6	6.5	7	5.5	5.5	6.5	6.1
SCaNP	17A	7.5	8	7	8	7.5	8	8	8.5	8	8	7.85
SCaNP	18C	6.5	6.5	6	6.5	6.5	6.5	7.5	7.5	7	7	6.75
SCaNP	18F	6.5	6.5	6.5	6.5	6	6.5	5.5	6.5	8	6.5	6.5
SCaNP	18G	4.5	5	4.5	4.5	5.5	5	4.5	4	5.5	4.5	4.75
SCaNP	18E	4.5	4.5	5.5	5	5.5	4.5	4.5	5.5	5.5	5.5	5.05
SCaNP	18B	6.5	6	7	6	7	7	6.5	6	6	6.5	6.45
SCaNP	18H	7	7.5	7	6.5	7.5	6.5	7	6.5	7.5	7	7
SCaNP	18A	7.5	6	6	8.5	7.5	8.5	5.5	8.5	6	5.5	6.95
SCaNP	18D	5.5	6	7	7	6.5	6.5	6	6.5	7	5.5	6.35
UNP	01A	5	6	5.5	5.5	5	5.5	5	6	5.5	4	5.3
UNP	02A	4.5	4.5	5	5	6	5	4.5	4.5	4.5	3	4.65
UNP	03A	4	3.5	4.5	5.5	5.5	5	4	4	4.5	4.5	4.5
UNP	04A	4.5	4	3.5	3.5	3.5	4	3	4	5	3	3.8
UNP	05A	5	6	5.5	5	5.5	6	5.5	6	6	5.5	5.6
UNP	06A	3.5	3	4	4	4.5	4.5	2.5	4	4	4	3.8
UNP	07A	3.5	4	4	4	4	3.5	4	3.5	4	4	3.85
UNP	08A	3.5	3.5	2	3.5	3.5	3	2	2	3	3.5	2.95
UNP	09A	4	4	4	4	3.5	4.5	5	3.5	4	3	3.95
UNP	10A	3.5	5.5	6	7	6	5	5	4	4	5	5.1
UNP	11A	5.5	3.5	4.5	4	3.5	4	4	5	4	4	4.2
UNP	12A	4.5	4	5	5.5	6	5	4.5	4.5	5	5	4.9
UNP	13A	7	5.5	6	7	5.5	4	4.5	5.5	4	6	5.5
UNP	14A	4	4	4	3.5	3.5	4.5	4.5	4.5	4	5	4.15
UNP	15A	6	5	5	5	6	5.5	6	5	5.5	7	5.6
UNP	16A	3	4	5.5	4.5	4.5	4	5.5	4.5	5.5	4.5	4.55
UNP	17A	5	5	4.5	4.5	4.5	4.5	5	6	5	4.5	4.85
UNP	18A	4	3	3.5	4	4	3	3.5	4	3.5	3.5	3.6
UNP	19A	4	4	4.5	4	3.5	3.5	7.5	4.5	5	4	4.45
UNP	20A	4.5	4.5	4	4.5	4	3.5	4	4	4	4.5	4.15
UNP	21A	5	5	4.5	5	5.5	8	5.5	6.5	5.5	6	5.65
UNP	22A	4	3	5	5	4	4.5	4	3	4	3.5	4
UNP	23A	3.5	4	5	4	4	3.5	4	4.5	5.5	4	4.2
UNP	24A	4.5	4.5	5	5	4	5	4	4.5	4.5	5.5	4.65
UNP	25A	4	3.5	4	3.5	4.5	4	4	4	4.5	3.5	3.95
UNP	26A	4	4	4	5	5	4.5	5	5	3.5	4	4.4
UNP	27A	7	7	6.5	7.5	8.5	5	6	5.5	7	7.5	6.75
UNP	28A	4.5	5	5.5	5.5	4.5	5	4.5	4.5	5.5	5	4.95
UNP	29A	4	4	3.5	5	4.5	3	4	3.5	6	4	4.15
UNP	30A	5.5	4.5	5	6.5	5.5	5	5	7	4.5	5.5	5.4
UNP	31A	5	5.5	5.5	6.5	5	4.5	5.5	3.5	6	5.5	5.25
UNP	32A	4.5	4.5	4.5	4	3.5	4.5	3	4	3.5	4.5	4.05
UNP	33A	7.5	6.5	8	7.5	6.5	4.5	6.5	6.5	7	6	6.65
UNP	34A	4.5	6.5	5	6	5	5.5	6.5	5	5.5	4.5	5.4
UNP	35A	4.5	6	5.5	4.5	6.5	5	5	4.5	4.5	5.5	5.15
UNP	36A	3.5	5.5	4.5	4	3.5	5.5	5	4.5	5.5	4	4.55
UNP	37A	4.5	5.5	7	4.5	4	4.5	4.5	4.5	4	3.5	4.65
UNP	38A	4	4	5	5.5	4	4.5	4	4	4	4.5	4.35
UNP	39A	4.5	4.5	5	6	4.5	4	5	4	5.5	4.5	4.75
UNP	40A	4	4.5	4	4	3.5	6.5	5.5	5.5	5	5	4.75
