# Supplementary material for: Wild capuchin monkeys use stones and sticks to access underground food
Source: Sci Rep. 2024 May 6;14:10415. doi: 10.1038/s41598-024-61243-8 (PMC11074112; doi:10.1038/s41598-024-61243-8)
Supplement: Supplementary file 2 — Supplementary Information 2. [file 41598_2024_61243_MOESM2_ESM.docx]

**Supplementary material**


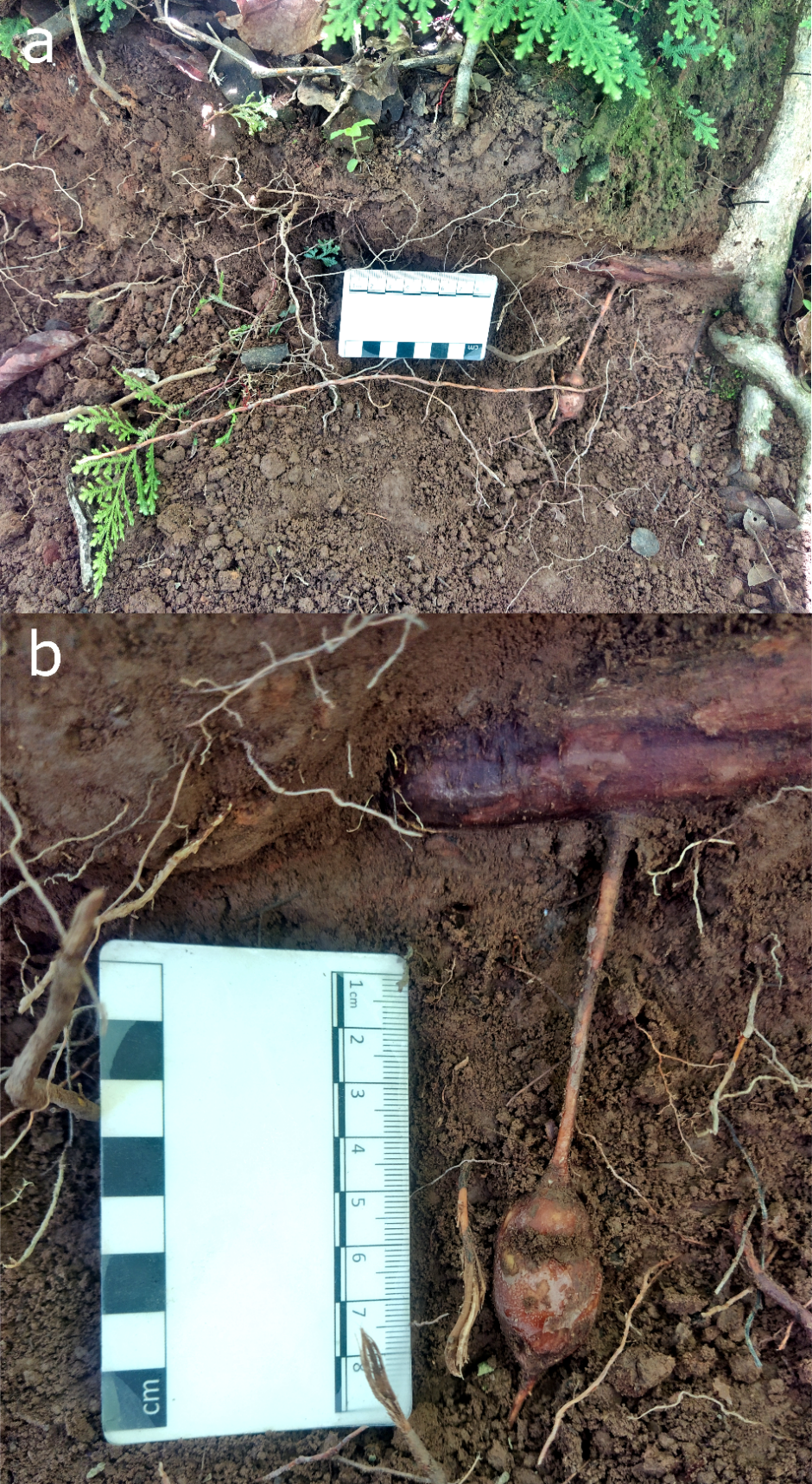


**Figure S1** – (a, b) Underground storage organ excavated by the researchers during the wet period in the same site excavated by a capuchin monkey. The roots of this plant species are reddish. Although we could not determine the species yet, it is morphologically similar to *farinha-seca* USOs (*Thiloa glaucocarpa*), excavated by capuchin monkeys in Serra da Capivara National Park.


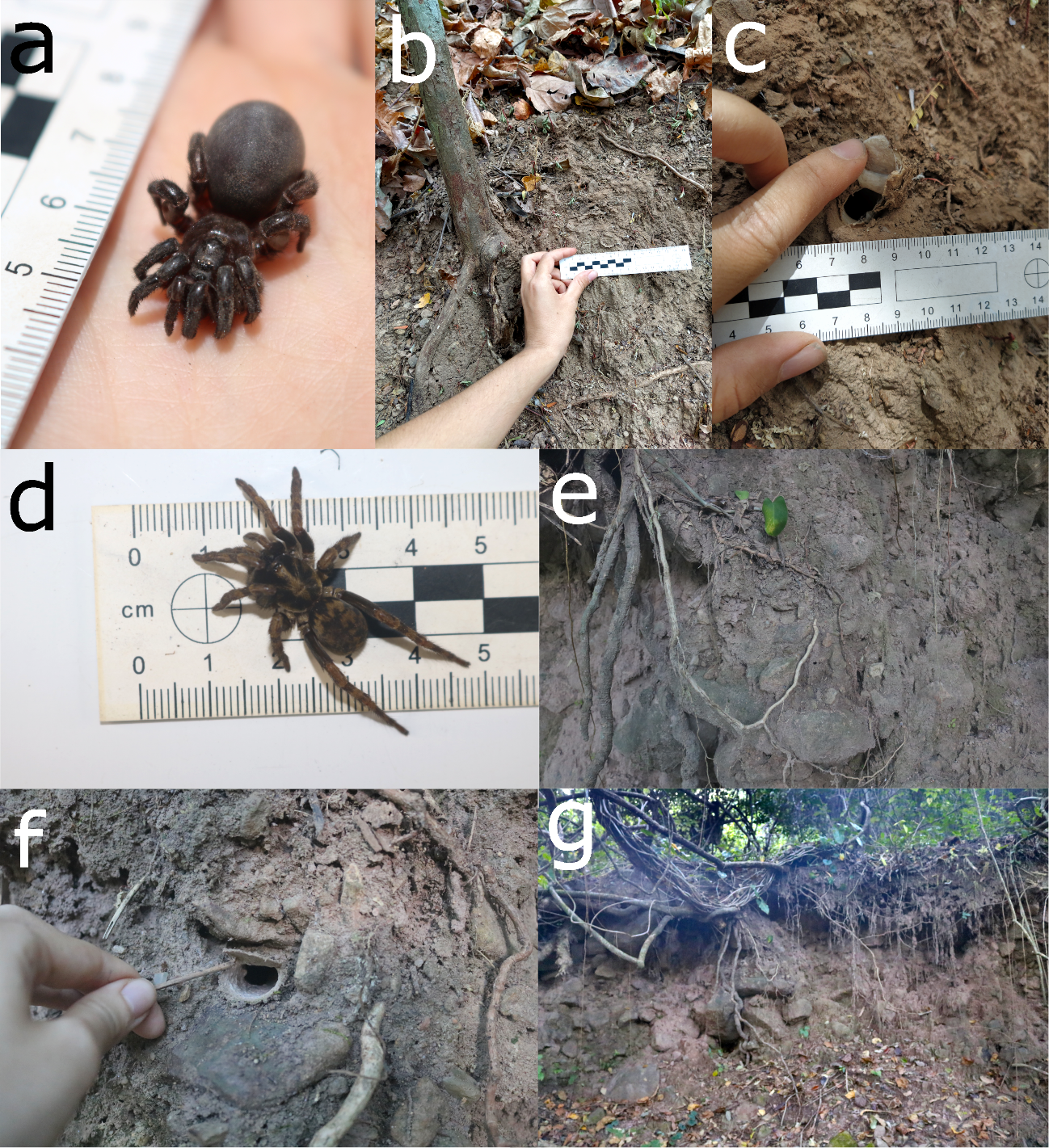


**Figure S2** – (a) Trapdoor spider of the species *Idiops sertania* (family Idiopidae) consumed by the capuchin monkeys in UNP; (b, c) its burrows, usually in ravines, with the trapdoor camouflaged in the surrounding ground; (d) Trapdoor spider of the species *Neodiplothele* sp. (family Barychelidae, species not described yet) consumed by the capuchin monkeys; (e) its morphology is less robust and they can lose some of their legs during the probing process (we find some legs inside the burrows after probing episodes); (f, g) and it also constructs burrows in ravines, especially in river banks, with the trapdoor camouflaged in the surrounding ground.


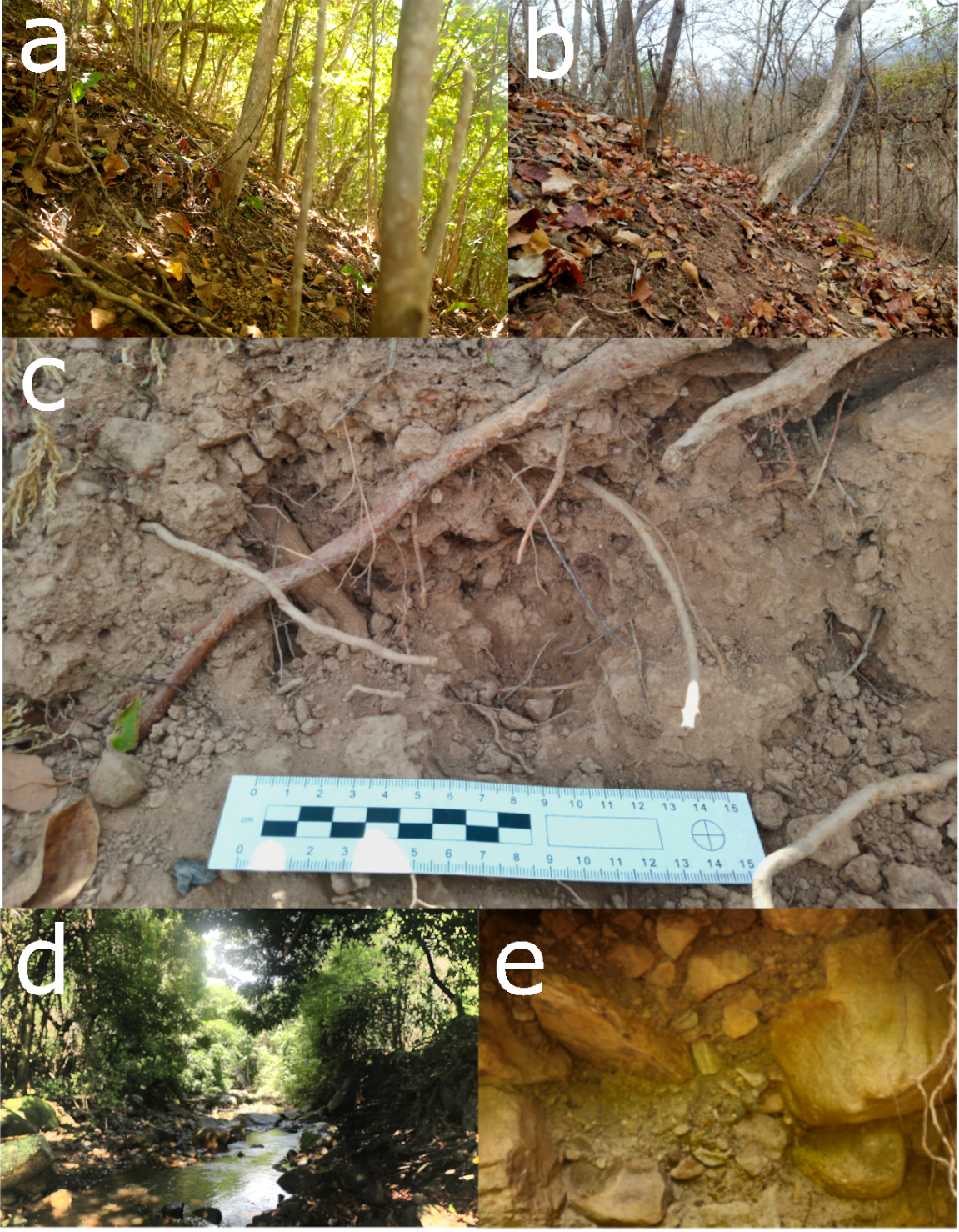


**Figure S3** - Ubajara’s hills are regions with elevated terrain, sparse and deciduous vegetation, and steep ground. The soil is stony and tough, especially during the dry season. Vegetation of the hills during the: (a) end of the wet season (camera is leveled and terrain inclination is ~37º); and (b) end of the dry season; (c) soil in the hills during the dry season where a subadult male successfully excavated an underground storage organ using a stone tool. Riverbanks are regions at the edge of rivers or streams (perennial or intermittent) with steep terrain. The soil is also stony and tough, especially because of embedded stones. Riverbanks (d) at the edges of the river; (e) with steep inclination; and full of embedded stones.

**Table S1 - GLM results for success in digging (with or without stones) and the predictor variables sex (males and females) and target (USOs and spider burrows), N= 127.** **Juveniles and unknown food targets were not included in this analysis.**

| **Success** | | | | |
| --- | --- | --- | --- | --- |
| **Effect** | **Estimate** | **SE** | **Z** | **p-value** |
| **Intercept** | 0.8091 | 0.4386 | 1.845 | 0.0651 |
| **Sex (Male)** | -0.3232 | 0.4370 | -0.739 | 0.4596 |
| **Target (USO)** | -0.9039 | 0.4157 | -2.175 | 0.0297* |

**Table S2 - Digging stone tools dimension (mean ± SD). N= 50. All sexes and ages are included.**

|  | **Weight (g)** | **Length (cm)** | **Width (cm)** | **Thickness (cm)** | **N** |
| --- | --- | --- | --- | --- | --- |
| ***USO*** | 138.6 ± SD 131.8 | 8.0 ± SD 3.0 | 5.3 ± SD 1.4 | 3.1 ± SD 0.8 | 33 |
| ***Spider burrow*** | 98.2 ± SD 49.2 | 7.1 ± SD 2.0 | 4.4 ± SD 1.3 | 2.9 ± SD 0.7 | 9 |
| ***Unknown*** | 121.3 ± SD 118.4 | 8.0 ± SD 2.5 | 5.2 ± SD 2.3 | 2.6 ± SD 0.4 | 8 |
| **Total** | 128.4 ± SD 117.9 | 7.9 ± SD 2.8 | 5.2 ± SD 1.5 | 2.9 ± SD 0.8 | 50 |

**Table S3 - Probing tools dimensions. N= 30.** **All sexes and ages are included.**

| **Site** | **Total length (cm)** | **Thickness (mm)** | **N** |
| --- | --- | --- | --- |
| **UNP** | 29.4 ± SD 12.6 | 2.9 ± SD 1.2 | 30 |
| **SCaNP** | 27.9 ± SD 14.97 | 4.7 ± SD 1.2 | 177 |

**Table S4 - GLM results for male success on foraging spider burrows and the predictor variable technique (hands-only, stick-probing, stone-digging, and stone-stick), N= 48. Females and juveniles were not included in this analysis.**

| **Male success** | | | | | |
| --- | --- | --- | --- | --- | --- |
|  | **Effect** | **Estimate** | **SE** | **Z** | **p-value** |
| **Technique** | Intercept | 1.609e+00 | 1.095e+00 | 1.469 | 0.1418 |
|  | Stick-probing | -1.920e+00 | 1.165e+00 | -1.648 | 0.0995 |
|  | Stone-digging | -2.015e+00 | 1.271e+00 | -1.585 | 0.1130 |
|  | Stone-Stick | 4.343e-15 | 1.549e+00 | 0.000 | 1.0000 |


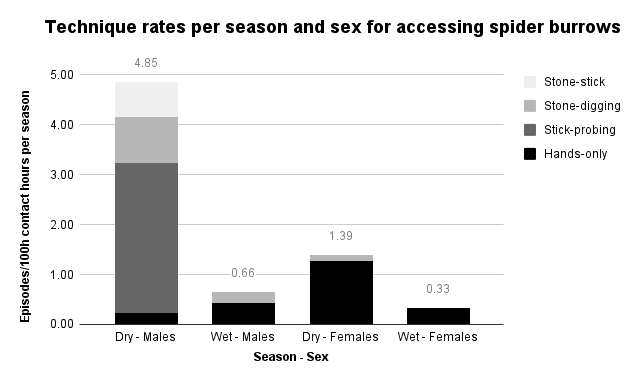


**Figure S4** – Number of episodes of foraging spider burrows using each technique in dry and wet season, considering the contact hours with the group in each season. Sexes are not comparable since we did not consider the contact time with each sex. Juveniles were not included. N= 63


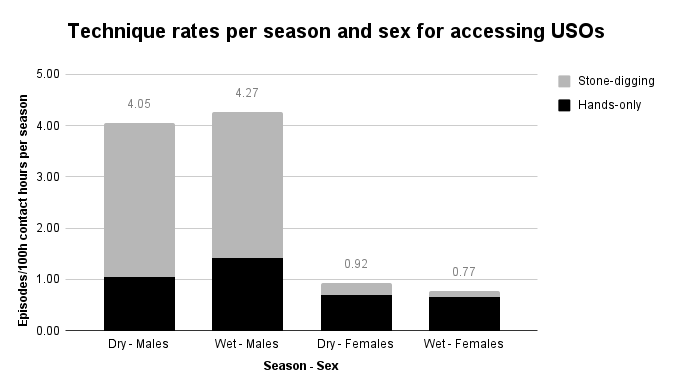


**Figure S5** – Number of episodes of foraging USOs using each technique in dry and wet season, considering the contact hours with the group in each season. Sexes are not comparable since we did not consider the contact time with each sex. Juveniles were not included. N= 89.


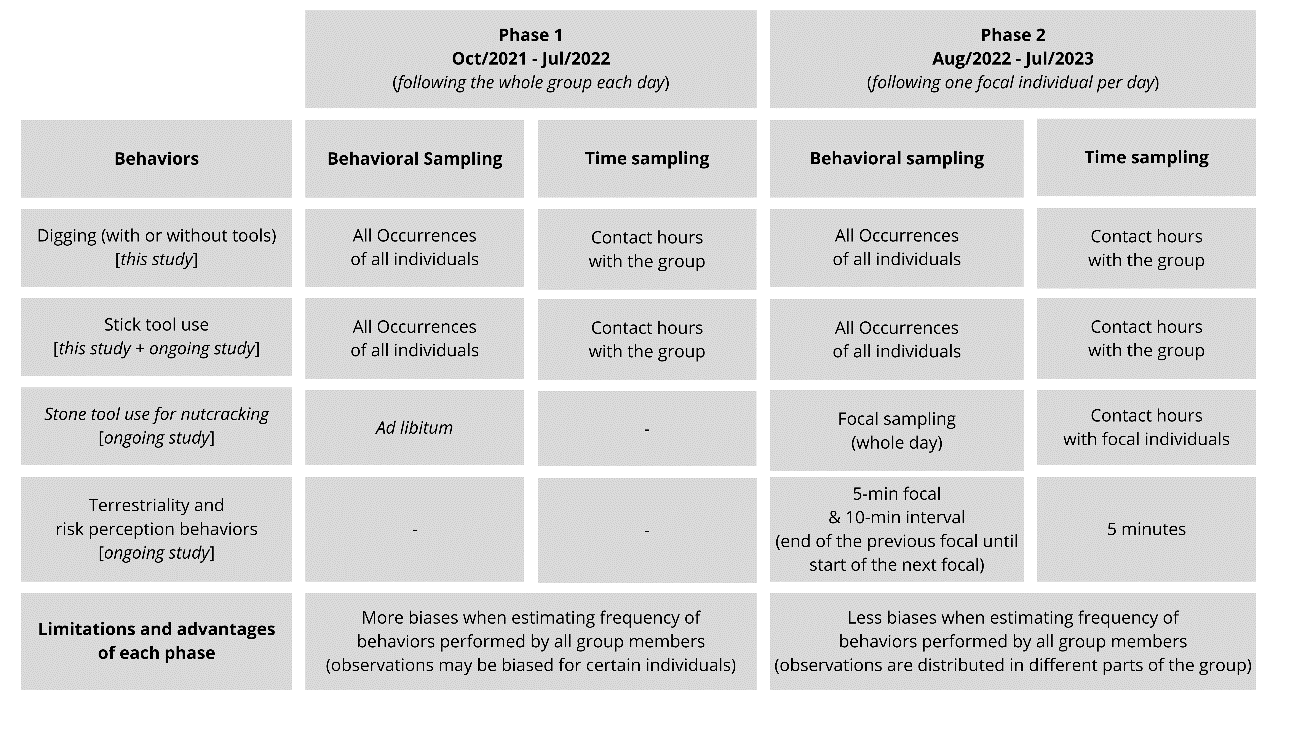


**Figure S6** – Data collection occurred in two phases. In the first phase (October/2021 to July/2022), we followed the whole group and in the second phase (August/2022 to July 2023), we systematically followed one adult a day. In both phases, we recorded digging behavior and the use of sticks by any individual of the group using All Occurrences sampling. It was possible during the second phase because two observers followed the focal individual, then even if one observer was recording a 5-minute focal sampling of other data (i.e., terrestriality and risk perception behaviors for ongoing studies), the other observer was available to record digging and stick tool use performed by any individual around the focal animal. Although we used the same sampling methods in both phases, the second phase had less bias because observations were distributed in different parts of the group.


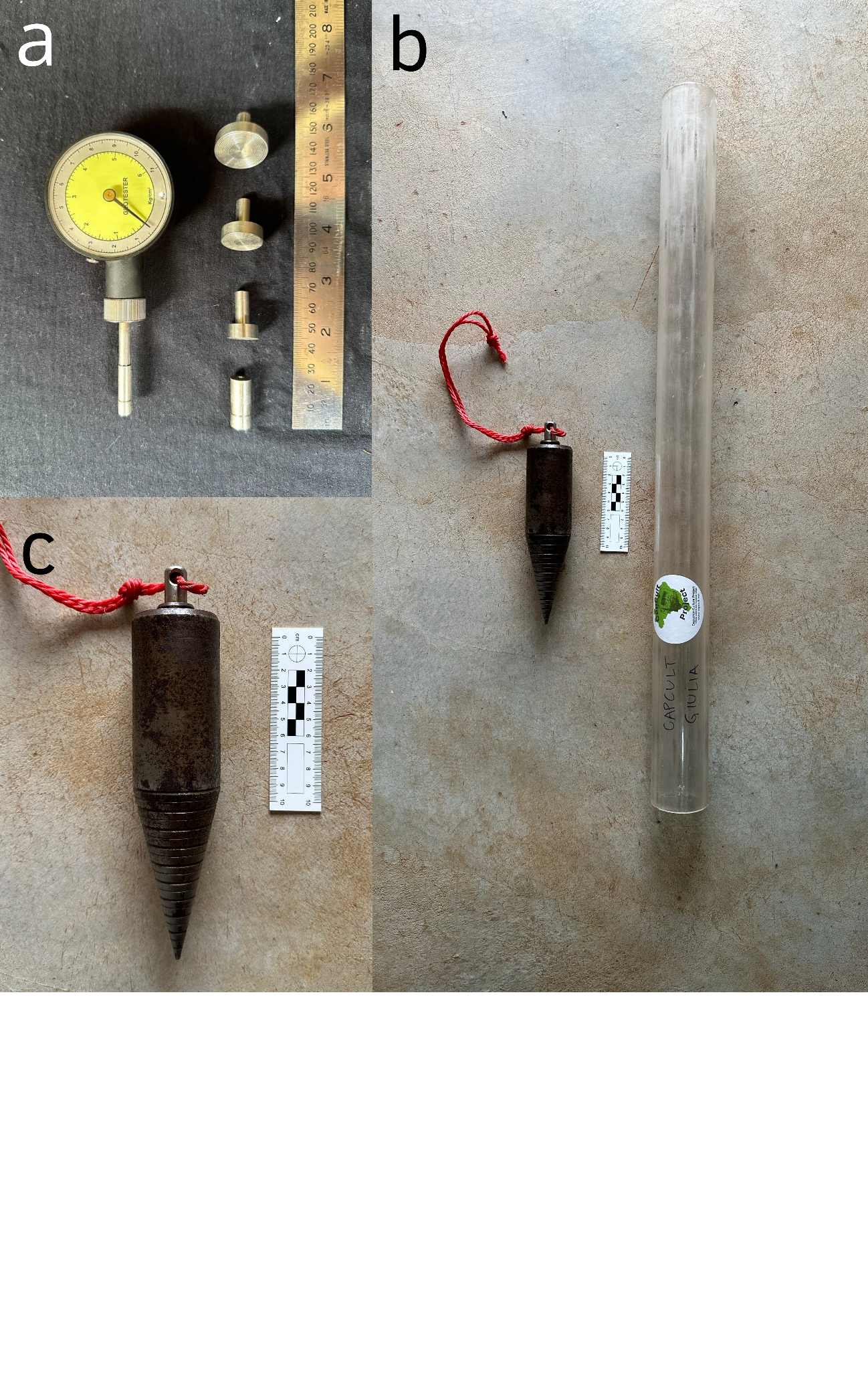


**Figure S7** – Equipment used for measuring absolute and relative soil cohesion: (a) pocket penetrometer with the smallest prod accoupled to it and the other prods on the right; (b) drop penetrometer, including the plexiglass tube and the conical metal weight; (c) conical metal weight with markings.

**Supplementary analysis**

The data presented in the manuscript is based on All Occurrences sampling of digging (with and without tools) and stick tool use for all group individuals during the two phases of this study (see Figure S6). This method is useful for sampling infrequent behaviors. However, it has some limitations since we may unconsciously oversample certain individuals over others. In this supplementary analysis, we present a subset of our sampling to address these limitations.

In the second phase of our study, we followed one focal individual per day (excluding juveniles). We calculated the contact hours in direct observation with each focal individual in this phase. Then, we extracted from our behavioral sampling the episodes that occurred with the focal individual while we were following it. Thus, this subsampling is equivalent to using the Animal Focal method for sampling these behaviors.

In Table S5, we present the raw data for each individual and technique. Although this method recorded few episodes, our sampling of foraging behavior of underground resources is relatively well distributed across individuals.

In Table S6, we present the rate of each technique for each focal individual (episodes/hour of contact). In Tables S7 and S8, we present the rates grouped by sex and by different ages of the males.

This supplementary analysis shows that our findings that females used digging stone tools in a lower proportion of their digging episodes than males is not a sampling bias. Females were more sampled, and we recorded many episodes of them using the hands-only technique for obtaining underground resources. However, stone-digging is so infrequent among them compared to males that we did not record any episode when using this sampling method. Our data also shows that subadult males explore more underground resources. However, adult males do not show a preference for hands-only (which has a higher general success) compared to stone-digging, and subadult and adult males present a similar rate of stick-probing.

Using the Focal Animal method for sampling infrequent behaviors also has limitations since we may have sampled an uncommon day that an individual used a lot of a technique and missed the use of other techniques that may have been more frequent in other days that it was not sampled. If we were using only Animal Focal, we would conclude that females do not use stone-digging technique, for example, which is not true. This technique also reduces sampling size, preventing us from analyzing differences in food targets, success, season, etc. Animal Focal is a challenging method to sample neotropical primates, which tend to have smaller body sizes and be arboreal, particularly in an environment that, despite being within a Caatinga biome, is much wetter than other savannah-like areas and presents a high vegetation density. Thus, we recommend that Focal Animal sampling should be used to sample the foraging behavior of underground resources only in long-term studies (which is the ideal method for calculating rates, but it has the disadvantages of being more expensive and taking more time).

**Table S5 – Time contact and episodes of different techniques for extraction of underground resources (hands-only, stone-digging, stone-stick, and stick-probing) by each focal individual (female adults: >5 years; male subadults: 5-7 years; male adults: >7 years).**

| **Individual** | **Sex** | **Age** | **Time contact (h)** | **Hands-only** | **Stone-digging** | **Stone-stick** | **Stick- probing** |
| --- | --- | --- | --- | --- | --- | --- | --- |
| Açucena | Female | Adult | 20.767 | 1 | 0 | 0 | 0 |
| Baião* | Male | Adult | 5.533 | 0 | 0 | 0 | 0 |
| Baleia | Female | Adult | 48.949 | 1 | 0 | 0 | 0 |
| Betânia | Female | Adult | 24.652 | 1 | 0 | 0 | 0 |
| Cajuína | Female | Adult | 45.864 | 2 | 0 | 0 | 0 |
| Canjica | Male | Subadult | 39.9 | 1 | 6 | 0 | 0 |
| Chicó | Male | Adult | 47.05 | 1 | 1 | 0 | 0 |
| Conceição | Female | Adult | 32.517 | 8 | 0 | 0 | 0 |
| Cordel* | Female | Adult | 2.217 | 0 | 0 | 0 | 0 |
| Cuscuz | Male | Adult | 41.717 | 0 | 0 | 0 | 0 |
| Fabiano | Male | Adult | 60.066 | 2 | 2 | 0 | 1 |
| João Grilo | Male | Subadult | 54.152 | 3 | 0 | 0 | 1 |
| Maria Bonita | Female | Adult | 57.066 | 1 | 0 | 0 | 0 |
| Maria Moura | Female | Adult | 47.867 | 3 | 0 | 0 | 0 |
| Mucunzá | Male | Subadult | 58.5 | 3 | 3 | 0 | 0 |
| Tapioca | Female | Adult | 35.432 | 1 | 0 | 0 | 0 |
| Xote | Female | Adult | 40.783 | 0 | 0 | 0 | 0 |

*Individuals who exited the group in the beginning of the second phase of this study.

**Table S6 – Technique rates (episodes/ hours of contact) for the extraction of underground resources (hands-only, stone-digging, stone-stick, and stick-probing) by each focal individual (female adults: >5 years; male subadults: 5-7 years; male adults: >7 years).**

| **Individual** | **Sex** | **Age** | **Hands-only rate** | **Stone-digging rate** | **Stone-stick rate** | **Stick-probing rate** |
| --- | --- | --- | --- | --- | --- | --- |
| Açucena | Female | Adult | 0.05 | 0.00 | 0.00 | 0.00 |
| Baião | Male | Adult | 0.00 | 0.00 | 0.00 | 0.00 |
| Baleia | Female | Adult | 0.02 | 0.00 | 0.00 | 0.00 |
| Betânia | Female | Adult | 0.04 | 0.00 | 0.00 | 0.00 |
| Cajuína | Female | Adult | 0.04 | 0.00 | 0.00 | 0.00 |
| Canjica | Male | Subadult | 0.03 | 0.15 | 0.00 | 0.00 |
| Chicó | Male | Adult | 0.02 | 0.02 | 0.00 | 0.00 |
| Conceição | Female | Adult | 0.25 | 0.00 | 0.00 | 0.00 |
| Cordel | Female | Adult | 0.00 | 0.00 | 0.00 | 0.00 |
| Cuscuz | Male | Adult | 0.00 | 0.00 | 0.00 | 0.00 |
| Fabiano | Male | Adult | 0.03 | 0.03 | 0.00 | 0.02 |
| João Grilo | Male | Subadult | 0.06 | 0.00 | 0.00 | 0.02 |
| Maria Bonita | Female | Adult | 0.02 | 0.00 | 0.00 | 0.00 |
| Maria Moura | Female | Adult | 0.06 | 0.00 | 0.00 | 0.00 |
| Mucunzá | Male | Subadult | 0.05 | 0.05 | 0.00 | 0.00 |
| Tapioca | Female | Adult | 0.03 | 0.00 | 0.00 | 0.00 |
| Xote | Female | Adult | 0.00 | 0.00 | 0.00 | 0.00 |

**Table S7 – Technique rates per sex (episodes/ hours of contact, juveniles excluded).**

| Sex | Time contact (h) | Hands-only | Stone-digging | Stone-stick | Stick probing |
| --- | --- | --- | --- | --- | --- |
| Females | 356.114 | 0.051 | 0.000 | 0.000 | 0.000 |
| Males | 306.918 | 0.033 | 0.039 | 0.000 | 0.007 |

**Table S8 – Technique rates used by males per age (episodes/ hours of contact, only males, juveniles excluded).**

| Age | Time contact (h) | Hands-only | Stone-digging | Stone-stick | Stick probing |
| --- | --- | --- | --- | --- | --- |
| Adult | 154.366 | 0.0194 | 0.0194 | 0.0000 | 0.0065 |
| Subadult | 152.552 | 0.0459 | 0.0590 | 0.0000 | 0.0066 |

**Supplementary legend**

**Video S1** – Examples of tools and techniques used by capuchin monkeys at Ubajara National Park for extracting underground storage organs and trapdoor spiders from the ground: digging with stone tools (‘stone-digging’), adjusting stone tool position, probing with stick tools (‘stick-probing’), and the use of a tool set (‘stone-stick’ technique).
